# Integrating Morpho-Physiological, Biochemical, and Molecular Genotyping for Selection of Drought-Tolerant Pigeon Pea (*Cajanus cajan* L.) Genotypes at Seedling Stage

**DOI:** 10.3390/plants13223228

**Published:** 2024-11-16

**Authors:** Benjamin O. Ouma, Kenneth Mburu, Geoffrey K. Kirui, Edward K. Muge, Evans N. Nyaboga

**Affiliations:** 1 Department of Biochemistry, University of Nairobi, P.O. Box 30197, Nairobi 00100, Kenya; bnjmnpk525@gmail.com (B.O.O.); mugeek@uonbi.ac.ke (E.K.M.); nyaboga@uonbi.ac.ke (E.N.N.); 2 Department of Life Sciences, South Eastern Kenya University, P.O. Box 170, Kitui 90200, Kenya; 3 Department of Biology, University of Nairobi, P.O. Box 30197, Nairobi 00100, Kenya; gkirui@uonbi.ac.ke

**Keywords:** biochemical assays, *Cajanus cajan*, drought tolerance, relative water content, SCoT, water stress

## Abstract

Pigeon pea (*Cajanus cajan* (L.) Millsp.), a potential legume as an economic source of protein, is commonly cultivated in tropical and subtropical regions of the world. It possesses medicinal properties and acts as a cash crop, benefiting low-income farmers economically. The identification of pigeon peas exhibiting drought tolerance has become crucial in addressing water scarcity issues in the agriculture sector. In addition, exploring the genetic diversity among genotypes is important for conservation, management of genetic resources, and breeding programs. The aim of this study was to evaluate the morpho-physiological and biochemical responses of selected pigeon pea genotypes under pot-induced water stress conditions through different field capacities as well as the genetic diversity using start codon targeted (SCoT) markers. A significant variation was observed for the physiological traits studied. The accumulation of fresh weight (FW) and dry weight (DW) was significantly reduced in moderate and severe drought stress conditions. The lowest % DW decrease was found in LM (35.39%), KAT (39.43%), and SM (46.98%) than other genotypes at severe drought stress. Analyses of physiological responses including the photosynthetic efficiency (Phi2), the chlorophyll content (SPAD), and the relative water content (RWC) revealed positive and negative correlations with various parameters, reflecting the impact of drought stress on the chlorophyll content. The results revealed that biochemical traits including the total phenolic content, soluble sugars, proline, total protein, total amino acids, and free amino acids were variably and significantly increased under water stress. Antioxidant enzyme activity levels, specifically ascorbate peroxidase (APX) and catalase, varied among the genotypes and in response to severe water stress, offering further insights into adaptive responses. The eight genotypes analysed by use of 20 SCoT markers revealed 206 alleles and an average of 10.3 alleles per locus. Genetic similarity ranged from 0.336 to 0.676, clustering the pigeon pea genotypes into two major groups by the unweighted pair group method of arithmetic averages (UPGMA) cluster analysis. Principal coordinate analysis (PCoA) explained 43.11% of genetic variation and based on analysis of molecular variance, a high genetic variation (80%) within populations was observed, emphasizing the potential for genetic improvement. Among the eight genotypes studied, LM and KAT were drought tolerant and genetically diverse and therefore could be used as parents for developing drought tolerance in pigeon pea.

## 1. Introduction

Pigeon pea (*Cajanus cajan* L.) is a perennial legume with global cultivation predominantly in tropical and semitropical regions. Globally, pigeon pea production is ranked sixth after dry bean, chickpea, field pea, cowpea, and lentil [1]. The South Asia region is the primary producer, contributing to more than 90% of the world’s pigeon pea production [1]. In Kenya, pigeon pea ranks third among food grain legumes, after common bean and cowpea [2]. The widespread popularity of pigeon pea among smallholder farmers can be attributed to its multifaceted utility. Notably, pigeon pea seeds are highly nutritious and serve as a crucial protein source, particularly for people in developing countries [3,4,5]. Mature pigeon pea seeds contain approximately 18.8% protein, 53% starch, 2.3% fat, 6.6% crude fibre, and 250.3 mg of minerals per 100 g [6]. Additionally, pigeon pea possesses medicinal properties due to the presence of various phytochemicals, including alkaloids (34%), flavonoids (46%), sterols (22%), and phenols (44%), contributing to its significant role in traditional medicine [7]. Pigeon pea plants offer fodder for domestic animals, materials for thatching and fencing, contribute to soil erosion control, and provide fuel wood [2,8]. Being a perennial shrub, pigeon pea offers advantages such as multiple harvests and significant contributions to soil fertility [2]. The crop boasts high biomass productivity primarily used as fodder, and enriches soil nutrient and moisture content [9,10]. Importantly, pigeon pea serves as a cash crop for low-income farmers, enhancing their economic stability [11]. However, the yield of pigeon pea is still low to the partial adaptability of the available genotypes to environmental stresses associated with climate change.

Climate change, particularly irregular rainfall and prolonged droughts, poses significant risks to crop development and growth [12]. Pigeon pea is known to exhibit drought tolerance, although drought stress significantly affects its development and growth at the seedling and early reproductive phases. Therefore, assessing drought stress tolerance in pigeon pea at the seedling stage is vital in the context of current and projected erratic precipitation patterns and prolonged droughts as a result of climate change [13]. Drought stress profoundly affects crop performance, triggering physiological and biochemical responses with intricate implications for growth and yield [14]. Reactive oxygen species (ROS) and reactive nitrogen species (RNS) generated under drought stress disrupt cellular redox regulatory functions, inducing oxidative stress [15,16]. The delicate balance between ROS production and the antioxidant defence system determines the cellular damage extent, impacting membrane integrity, protein structure, and nucleic acid stability [17,18,19].

Oxidative stress adversely affects plant health, growth, and development, causing lipid peroxidation, enzyme inactivation, and DNA damage [18,19]. Elevated ROS levels under drought stress compromise membrane integrity, disrupting nutrient uptake and transport, and alter protein structure, leading to enzyme inactivation [18,19]. These impacts extend to nucleic acids, with ROS-induced DNA damage affecting genetic stability [17,18,19]. Oxidative stress inhibits cell division and elongation, resulting in stunted growth and a reduced leaf area in plants facing water deficit conditions [18,20]. These alterations collectively compromise reproductive development in crops enduring prolonged water deficit, adversely affecting yield components such as grain number and size, impacting economic returns for farmers [21]. Stomatal closure, a fundamental water conservation mechanism, limits carbon dioxide influx for photosynthesis, reducing energy production and altering carbon partitioning [22]. This contributes to reduced photosynthetic activity, coupled with membrane injury and altered enzyme functioning, especially associated with ATP synthesis [16]. Hormonal signalling, notably involving abscisic acid (ABA), plays a crucial role in regulating drought response mechanisms [23,24]. Prolonged drought disrupts hormonal homeostasis, profoundly impacting overall plant growth and development [20,24,25]. Drought-induced outcomes include decreased cell division, compromised root differentiation, altered foliage dimensions, reduced shoot length, and modified stomatal movements, collectively diminishing water and mineral nutrition associations, ultimately decreasing the plant yield and water usage efficacy [25]. Given the global challenge of sustaining food production under changing climatic conditions, these physiological alterations underscore the urgent need for drought-resilient crops.

The success of breeding programs in any crop relies on the genetic variability among the genotypes available to breeders for developing desirable varieties [26]. They are crucial for assessing the availability and variability of target traits in breeding programs [27,28,29]. Genetic variation is paramount for effective breeding and germplasm conservation of pigeon pea genotypes [30,31,32]. Exploring genetic variations within pigeon pea genotypes can lead to the development of improved cultivars that are more resilient to environmental stress and better suited to the diverse needs of farmers and consumers [30,33]. Therefore, assessing genetic variation is the foundation in plant breeding as it enables the selection of varieties that exhibit desirable traits for crop improvement programs [34,35]. Molecular markers have been reported to be effective in the determination of the genetic variation of crop plants [36,37]. The start codon targeted (SCoT) marker is one of the molecular markers and exclusively targets the gene loci bordering the translation start codon (i.e., ATG) on both sense and antisense strands of DNA [38]. The start codon targeted marker–based system has distinct advantages over other widely used molecular markers such as SSR, RAPD, ISSR, and AFLP. The advantages include its simplicity requiring a single primer for amplification, cost-effectiveness, high reproducibility, high polymorphism, and extensive genetic information [39,40,41,42,43]. Notably, it exhibits universality across various plant species. These features render SCoT marker–based genotyping an efficient and versatile tool for studying trait-associated variations in pigeon pea.

In this study, we hypothesize (Null; Ho) that variations are absent in morphological, physiological, and biochemical responses of pigeon pea genotypes to drought stress against the Alternate Hypothesis (H1) that variations exist in responses of pigeon pea genotypes to drought stress as may be revealed in morphological, physiological, and biochemical traits. We thus report the evaluation of the response of eight genotypes of pigeon pea to drought stress treatments, with the overall aim of identifying pigeon genotypes with the potential as drought-tolerant hybrids.

## 2. Results

### 2.1. Effects of Drought on Growth and Biomass Production at the Seedling Stage

The mean growth and biomass production parameters are presented in Table 1. Drought stress significantly (*p* < 0.05) inhibited the growth of pigeon pea seedlings and reduced the fresh and dry weights of plants relative to the well-watered plants. Drought stress significantly (*p* < 0.05) reduced the root and shoot lengths (Table 1). The drought-induced effect was significantly (*p* ˂ 0.05) higher in severely stressed plants as compared to moderately stressed plants for the pigeon pea genotypes (Table 1). The accumulation of fresh weight (FW) and dry weight (DW) was significantly reduced in the moderate and severe drought stress conditions (Table 1). The lowest % DW decrease was found in LM (35.39%), KAT (39.43%), and SM (46.98%) than other genotypes at severe drought stress. The % of DW accumulation was low in the P1 (38.97%) and P9 (43.61%) genotypes at severe drought stress conditions.

### 2.2. Effects of Drought on Physiological Traits of Pigeon Pea Genotypes at the Seedling Stage

The effect of drought stress treatments on photosynthetic parameters namely Phi2 (Quantum yield of Photosystem II), PhiNPQ (Photochemical quenching), FvP/FmP (Quantum efficiency of Photosystem II), linear electron flow (LEF), non-photochemical quenching (NPQt), non-regulatory energy dissipation (PhiNO), relative chlorophyll content (SPAD), and leaf temperature is presented in Table 2. Under water stress conditions, plants of all pigeon pea genotypes exhibited a consistent decline in photosynthetic parameters as compared to their well-watered (100% field capacity) plants (Table 2 and Table S1).

**Table 1:** Growth and biomass production parameters in pigeon pea.

	Shoot Length (SL; cm)			Root Length (RL; cm)			Fresh Weight (FW; g per Plant)			Dry Weight (DW; g per Plant)		
Genotype	FC	50% FC	25% FC	FC	50% FC	25% FC	FC	50% FC	25% FC	FC	50% FC	25% FC
P1	25.14 ± 0.26 ^a^	16.92 ± 0.27 ^b^	12.91 ± 0.24 ^b^	23.38 ± 0.53 ^a^	18.21 ± 0.23 ^a^	11.97 ± 0.02 ^c^	32.43 ± 1.76 ^a^	26.86 ± 1.11 ^b^	23.15 ± 0.80 ^b^	11.16 ± 0.74 ^b^	7.11 ± 0.68 ^b^	4.35 ± 0.11 ^c^
P2	24.19 ± 0.53 ^a^	16.48 ± 0.19 ^b^	13.72 ± 0.44 ^b^	21.17 ± 0.36 ^b^	16.14 ± 0.52 ^ab^	12.18 ± 0.01 ^c^	33.48 ± 0.90 ^a^	28.05 ± 1.22 ^ab^	24.92 ± 1.11 ^ab^	12.63 ± 0.13 ^ab^	7.14 ± 0.22 ^b^	5.91 ± 0.56 ^bc^
P3	25.33 ± 0.56 ^a^	16.54 ± 0.23 ^b^	12.75 ± 0.18 ^b^	19.98 ± 0.37 ^b^	15.17 ± 0.42 ^b^	11.63 ± 0.01 ^c^	33.47 ± 1.23 ^a^	29.32 ± 1.44 ^a^	24.62 ± 1.07 ^ab^	11.12 ± 0.91 ^b^	7.62 ± 0.55 ^b^	5.32 ± 0.81 ^bc^
SM	26.22 ± 0.41 ^a^	17.76 ± 0.34 ^b^	14.10 ± 0.53 ^b^	20.02 ± 0.15 ^b^	17.28 ± 0.55 ^a^	14.75 ± 0.005 ^a^	32.63 ± 0.88 ^a^	30.13 ± 1.08 ^a^	23.51 ± 0.76 ^b^	13.56 ± 0.54 ^a^	9.53 ± 0.31 ^a^	7.19 ± 0.61 ^b^
MM	26.24 ± 0.23 ^a^	17.05 ± 0.41 ^b^	14.43 ± 0.73 ^b^	19.57 ± 0.41 ^b^	15.11 ± 0.81 ^b^	12.13 ± 0.002 ^c^	33.34 ± 0.97 ^a^	28.50 ± 0.71 ^ab^	23.26 ± 0.96 ^b^	12.43 ± 0.51 ^ab^	8.41 ± 0.57 ^b^	5.42 ± 0.71 ^bc^
LM	24.92 ± 0.36 ^a^	19.92 ± 0.38 ^a^	15.93 ± 0.91 ^a^	19.98 ± 0.81 ^b^	17.40 ± 0.13 ^a^	15.16 ± 0.003 ^a^	34.71 ± 1.07 ^a^	30.79 ± 1.10 ^a^	26.35 ± 0.85 ^a^	13.76 ± 0.38 ^a^	10.73 ± 0.48 ^a^	8.79 ± 0.62 ^a^
KAT	25.41 ± 0.73 ^a^	20.18 ± 0.51 ^a^	16.28 ± 0.83 ^a^	20.48 ± 0.43 ^b^	17.23 ± 0.42 ^a^	14.97 ± 0.01 ^a^	33.45 ± 1.19 ^a^	31.18 ± 0.91 ^a^	25.72 ± 1.19 ^a^	14.81 ± 0.38 ^a^	11.86 ± 0.52 ^a^	8.97 ± 0.33 ^a^
P9	25.97 ± 0.15 ^a^	16.22 ± 0.23 ^b^	13.49 ± 0.57 ^b^	21.38 ± 0.33 ^b^	15.98 ± 0.57 ^b^	13.15 ± 0.004 ^ab^	34.23 ± 1.08 ^a^	26.87 ± 0.42 ^b^	22.98 ± 1.22 ^b^	11.19 ± 0.42 ^b^	7.47 ± 0.33 ^b^	4.88 ± 0.52 ^c^

Values represent means with standard errors (n = 5). In the same column, different lowercase letters represent significant alterations among pigeon pea genotypes within the same stress level, according to Scott–Knott’s test (*p* < 0.05).

Analysis of variance (ANOVA) was conducted to assess the impact of different factors on various physiological traits in this study (Supplementary Table S2). Genotypes (G) significantly influenced all the physiological traits studied (Phi2, PhiNPQ, PhiNO, SPAD, LEF, NPQt, FvP/FmP, and LT), as indicated by the highly significant *p*-values (*p* < 0.001) and large mean-square values. The water regime (W) also had a significant impact on all the physiological traits, with highly significant *p*-values (*p* < 0.001) and large mean-square values (Supplementary Table S2). The level of water availability significantly affected the physiological responses of plants for all the pigeon pea genotypes (Table 2).

### 2.3. Relative Water Content

The relative water content (RWC) of the eight pigeon pea genotypes was assessed under different water stress levels. At 100% FC, the RWC ranged from 73.02% to 89.6%, with genotypes P3 and MM having the lowest (73.02%) and highest (89.6%), respectively. Under 50% FC, there was significant decrease in the RWC, with the drought-tolerant genotypes LM and KAT having significantly (*p* ≤ 0.05) high RWC. At 25% FC, the RWC decreased further (62.22% to 78.26%), with genotypes P3 and KAT having the lowest and highest, respectively (Table 2). In general, there was significant (*p* ≤ 0.05) decrease in the RWC in the individual genotypes as the severity of drought stress increased (Table 2).

### 2.4. Effect of Water Stress on Oxidative Stress Markers

Under moderate and severe drought-stressed conditions, lipid peroxidation (MDA) significantly enhanced as compared to normal irrigated conditions in all investigated pigeon pea genotypes. Under 50% FC, MDA content significantly increased for most genotypes, indicating elevated lipid peroxidation (Table 3). Genotypes P3 and P1 had the highest (8.46 nm g^−1^ FW), and lowest (1.12 nm g^−1^ FW) increase in MDA content. At 25% FC, MDA content further increased, indicating more pronounced lipid peroxidation due to water stress. Short-maturing (SM), MM, LM, KAT, and P9 showed a similar trend of increasing MDA content with decreasing water availability, with the highest at 25% FC. In general, lipid peroxidation increased as the availability of water decreased (Table 3).

Under FC conditions, H_2_O_2_ content varied among the pigeon pea genotypes, with a significantly (*p* ≤ 0.05) high concentration (3.05 μmol/gFW) exhibited by genotype P2 as compared with other genotypes. At 50% FC, H_2_O_2_ content showed minor fluctuations among genotypes, with genotype P2 having the highest content at 3.22 μmol/gFW, and genotype P1 showed a slight increase to 2.81 μmol/gFW (Table 2). Under severe water stress conditions (25% FC), H_2_O_2_ content also varied, with genotype P1 having the highest content at 3.67 μmol/gFW, while for genotype P2, the content decreased to 2.67 μmol/gFW (Table 3).

### 2.5. Effect of Water Stress on Antioxidant Enzyme Activities

Under the different water stress treatments, the pigeon pea genotypes exhibited variations in total peroxidase (POD) activity. Under drought stress conditions, POD significantly increased in all the genotypes. In control conditions, genotype P1 had the highest activity at 49.20 mole/s, while genotype MM had the lowest at 19.18 mole/s. At 50% FC, POD activity fluctuated, with genotype P1 decreasing to 32.85 mole/s and genotype P3 increasing to 35.55 mole/s. Severe water stress (25% FC) resulted in varied activity, with genotype P1 decreasing to 28.92 mole/s and genotype SM increasing to 30.45 mole/s. The MM and LM genotypes maintained consistent levels, with a slight increase at 25% FC.

**Table 2:** Effect of water stress on physiological parameters of pigeon pea genotypes under glasshouse conditions.

Mean of Each Pigeon Pea Genotype for Physiological Traits Under Different Water Regimes															
	Quantum Yield of Photosystem II (Phi2)			PhiNPQ			PhiNO			SPAD			RWC		
Genotype	FC	50% FC	25% FC	FC	50% FC	25% FC	FC	50% FC	25% FC	FC	50% FC	25% FC	FC	50% FC	25% FC
P1	0.60 ± 0.01 ^ab^	0.53 ± 0.02 ^abc^	0.39 ± 0.01 ^abc^	0.18 ± 0.01 ^b^	0.25 ± 0.02 ^bc^	0.44 ± 0.03 ^cd^	0.21 ± 0.01 ^a^	0.21 ± 0.03 ^a^	0.17 ± 0.02 ^ab^	41.93 ± 2.76 ^ab^	32.86 ± 1.31 ^bc^	33.75 ± 1.48 ^ab^	80.6 ± 2.14 ^a^	79.21 ± 3.08 ^b^	68.35 ± 3.11 ^c^
P2	0.59 ± 0.008 ^b^	0.40 ± 0.08 ^c^	0.34 ± 0.01 ^c^	0.24 ± 0.02 ^b^	0.46 ± 0.09 ^a^	0.53 ± 0.02 ^b^	0.17 ± 0.01 ^b^	0.14 ± 0.01 ^d^	0.14 ± 0.01 ^cd^	44.46 ± 0.96 ^a^	30.05 ± 1.12 c	29.82 ± 2.08 ^b^	74.6 ± 2.33 ^a^	72.24 ± 3.00 ^b^	65.49 ± 1.92 ^c^
P3	0.50 ± 0.04 ^c^	0.50 ± 0.04 b^c^	0.22 ± 0.03 ^d^	0.33 ± 0.04 ^a^	0.33 ± 0.04 ^ab^	0.65 ± 0.04 ^a^	0.17 ± 0.01 ^b^	0.17 ± 0.01 ^cd^	0.13 ± 0.01 ^d^	37.67 ± 2.23 ^bc^	30.32 ± 3.44 c	34.26 ± 2.57 ^ab^	73.02 ± 1.91 ^a^	70.16 ± 3.55 ^b^	62.22 ± 1.82 ^c^
SM	0.60 ± 0.008 ^ab^	0.63 ± 0.02 ^ab^	0.35 ± 0.05 ^bc^	0.22 ± 0.01 ^b^	0.16 ± 0.02 ^c^	0.50 ± 0.05 ^bc^	0.18 ± 0.01 ^b^	0.20 ± 0.005 ^ab^	0.15 ± 0.005 ^bcd^	40.83 ± 0.85 ^ab^	36.13 ± 1.8 ab	37.11 ± 2.16 ^a^	79.56 ± 2.34 ^a^	78.3 ± 1.81 ^b^	72.19 ± 3.01 ^b^
MM	0.59 ± 0.006 ^b^	0.65 ± 0.02 ^a^	0.44 ± 0.03 ^a^	0.24 ± 0.01 ^b^	0.15 ± 0.01 ^c^	0.40 ± 0.03 ^d^	0.17 ± 0.01 ^b^	0.20 ± 0.008 ^abc^	0.16 ± 0.002 ^abc^	34.04 ± 0.57 ^c^	34.50 ± 0.75 ^abc^	37.6 ± 1.96 ^a^	89.6 ± 2.51 ^a^	79.48 ± 2.67 ^b^	73.72 ± 2.91 ^b^
LM	0.60 ± 0.02 ^ab^	0.51 ± 0.09 ^bc^	0.46 ± 0.003 ^a^	0.22 ± 0.04 ^b^	0.29 ± 0.08 ^bc^	0.38 ± 0.01 ^d^	0.18 ± 0.01 ^b^	0.20 ± 0.003 ^abc^	0.16 ± 0.003 ^abc^	40.72 ± 1.7 ^ab^	40.15 ± 2.10 a	38.65 ± 1.25 ^a^	86.96 ± 2.38 ^a^	84.13 ± 1.89 ^a^	70.69 ± 2.12 ^b^
KAT	0.61 ± 0.09 ^ab^	0.64 ± 0.01 ^ab^	0.44 ± 0.02 ^a^	0.21 ± 0.02 ^b^	0.16 ± 0.01 ^c^	0.38 ± 0.01 ^d^	0.18 ± 0.01 ^b^	0.20 ± 0.02 ^abc^	0.18 ± 0.01 ^a^	41.55 ± 3.69 ^ab^	36.68 ± 0.41 ab	35.76 ± 1.09 ^a^	86.96 ± 1.88 ^a^	84.96 ± 1.02 ^a^	78.26 ± 1.43 ^a^
P9	0.64 ± 0.01 ^a^	0.60 ± 0.02 ^ab^	0.42 ± 0.02 ^ab^	0.17 ± 0.02 ^b^	0.22 ± 0.03 ^bc^	0.43 ± 0.02 ^cd^	0.18 ± 0.01 ^b^	0.18 ± 0.005 ^bc^	0.15 ± 0.004 ^bcd^	41.28 ± 1.78 ^ab^	36.87 ± 2.32 ab	35.08 ± 1.20 ^ab^	81.12 ± 3.02 ^a^	78.87 ± 2.81 ^b^	74.18 ± 3.02 ^b^
Mean of each pigeon pea genotype for physiological traits under different water regimes															
	LEF			NPQt			FvP/FmP			Leaf temperature					
Genotype	FC	50% FC	25% FC	FC	50% FC	25% FC	FC			50% FC		25% FC	FC	50% FC	25% FC
P1	40.23 ± 4.22 ^ab^	39.9 ± 1.65 ^b^	47.4 ± 2.14 ^ab^	0.86 ± 0.04 ^b^	1.35 ± 0.35 ^bc^	2.8 ± 0.33 ^bc^	0.72 ± 0.005 ^a^			0.69 ± 0.02 ^ab^		0.58 ± 0.02 ^ab^	29.42 ± 0.1 ^h^	29.57 ± 0.28 ^e^	31.7 ± 0.29 ^c^
P2	31.45 ± 1.87 ^bc^	40.82 ± 1.89 ^b^	37.3 ± 5.44 ^b^	1.45 ± 0.17 ^b^	3.79 ± 1.00 ^a^	6.7 ± 2.73 ^a^	0.67 ± 0.01 b^c^			0.54 ± 0.05 ^c^		0.50 ± 0.02 ^cd^	31.37 ± 0.1 ^d^	31.18 ± 0.25 ^d^	35.01 ± 0.16 ^b^
P3	49.14 ± 4.86 ^a^	44.53 ± 4.28 ^b^	55.2 ± 10.2 ^ab^	2.25 ± 0.47 ^a^	2.36 ± 0.47 ^b^	5.16 ± 0.38 ^ab^	0.63 ± 0.02 ^c^			0.62 ± 0.02 ^b^		0.46 ± 0.03 ^d^	32.89 ± 0.05 ^c^	32.31 ± 0.16 b^c^	37.00 ± 0.23 ^a^
SM	37.14 ± 1.43 ^b^	38.05 ± 5.89 ^b^	61.71 ± 3.14 ^ab^	1.25 ± 0.17 ^b^	0.81 ± 0.08 ^c^	3.71 ± 0.50 ^abc^	0.69 ± 0.01 ^ab^			0.73 ± 0.09 ^a^		0.54 ± 0.03 ^bc^	33.9 ± 0.1 ^a^	32.65 ± 0.10 ^abc^	37.41 ± 0.08 ^a^
MM	39.74 ± 4.2 ^ab^	28.88 ± 3.94 ^b^	47.3 ± 6.53 ^ab^	1.56 ± 0.19 ^ab^	0.77 ± 0.02 ^c^	2.91 ± 0.14 ^bc^	0.67 ± 0.01 ^bc^			0.73 ± 0.02 ^a^		0.6 ± 0.01 ^ab^	33.46 ± 0.1 ^b^	32.06 ± 0.09 ^c^	37.06 ± 0.22 ^a^
LM	23.38 ± 0.9 ^c^	77.1 ± 27.3 ^a^	39.77 ± 3.55 ^b^	1.30 ± 0.34 ^b^	1.51 ± 0.47 ^bc^	3.63 ± 0.36 ^bc^	0.69 ± 0.03 ^ab^			0.68 ± 0.04 ^ab^		0.6 ± 0.01 ^ab^	30.23 ± 0.1 ^g^	32.20 ± 0.06 ^c^	35.55 ± 0.15 ^b^
KAT	22.46 ± 6.2 ^c^	28.09 ± 5.56 ^b^	35.94 ± 8.49 ^b^	1.19 ± 0.19 ^b^	0.80 ± 0.04 ^c^	2.13 ± 0.12 ^c^	0.69 ± 0.02 ^ab^			0.73 ± 0.04 ^a^		0.62 ± 0.01 ^a^	30.68 ± 0.1 ^f^	33.02 ± 0.49 ^ab^	30.75 ± 0.10 ^d^
P9	23.33 ± 2.24 ^c^	37.58 ± 4.83 ^b^	72.8 ± 20.5 ^a^	0.996 ± 0.16 ^b^	1.36 ± 0.20 ^bc^	2.89 ± 0.25 ^bc^	0.71 ± 0.01 ^ab^			0.68 ± 0.02 ^ab^		0.56 ± 0.01 ^ab^	31.05 ± 0.05 ^e^	33.16 ± 0.21 ^a^	32.25 ± 0.3 ^c^

Values are the mean (±S.E.) of five replicates, and different superscript letters indicate significant differences (*p* < 0.05). FC—Field capacity; Phi2—Quantum yield of Photosystem II; PhiNPQ—Photochemical quenching; FvP/FmP—quantum efficiency of Photosystem II (PSII); LEF—linear electron flow, NPQt—non-photochemical quenching; PhiNO—Non-regulatory energy dissipation; RWC—Relative water content. Values are means ± SD of three measurements for each of the three replicates. In the same column, different lowercase letters represent significant alterations among pigeon pea genotypes within the same stress level, according to Scott–Knott’s test (*p* < 0.05).

**Table 3:** Effect of drought stress on biochemical components of pigeon pea genotypes under glass house conditions. FC—100% field capacity, 50% FC—50% field capacity, 25% FC—25% field capacity. Genotypes: P1, P2, P3, SM, MM, LM, KAT, and P9.

Mean of Each Pigeon Pea Genotype for Biochemical Traits Under Different Water Regimes												
	MDA			H_2_O_2_			POD			CAT		
Genotype	FC	50% FC	25% FC	FC	50% FC	25% FC	FC	50% FC	25% FC	FC	50% FC	25% FC
P1	1.01 ± 0.25 ^a^	1.12 ± 1.01 ^c^	5.65 ± 0.79 ^c^	2.26 ± 0.1 ^b^	2.81 ± 0.47 ^a^	3.67 ± 0.11 ^a^	49.2 ± 3.54 ^a^	32.85 ± 1.72 ^a^	28.92 ± 2.37 ^a^	1.10 ± 0.02 ^a^	1.58 ± 0.11 ^a^	0.68 ± 0.08 ^a^
P2	2.86 ± 0.45 ^a^	4.86 ± 1.28 ^b^	12.52 ± 0.81 ^a^	3.05 ± 0.41 ^a^	3.22 ± 0.93 ^a^	2.67 ± 0.143 ^a^	41.08 ± 3.95 ^b^	38.53 ± 1.47 ^b^	11.99 ± 2.01 ^c^	0.33 ± 0.08 ^b^	0.45 ± 0.05 ^a^	0.33 ± 0.09 ^a^
P3	1.94 ± 0.52 ^a^	8.46 ± 1.33 ^a^	10.59 ± 0.49 ^a^	2.84 ± 0.55 ^a^	2.85 ± 0.87 ^a^	2.91 ± 0.22 ^a^	26.67 ± 3.44 ^c^	35.55 ± 1.68 ^b^	19.47 ± 2.93 ^b^	0.68 ± 0.12 ^b^	0.58 ± 0.01 ^a^	0.85 ± 0.03 ^a^
SM	2.10 ± 0.22 ^a^	5.13 ± 1.51 ^a^	10.59 ± 0.38 ^a^	2.47 ± 0.92 ^b^	2.73 ± 0.54 ^a^	2.85 ± 0.31 ^a^	22.57 ± 4.04 ^c^	29.95 ± 2.08 ^b^	30.45 ± 2.88 ^a^	0.35 ± 0.08 ^b^	1.25 ± 0.03 ^a^	0.93 ± 0.03 ^a^
MM	2.18 ±0.23 ^a^	6.64 ± 0.91 ^a^	8.14 ± 0.94 ^b^	2.73 ± 0.74 ^a^	2.81 ± 0.71 ^a^	3.05 ± 0.56 ^a^	19.18 ± 2.598 ^c^	25.89 ± 2.14 ^c^	30.98 ± 2.94 ^a^	0.15 ± 0.01 ^c^	0.68 ± 0.01 ^a^	0.78 ± 0.04 ^a^
LM	1.76 ±0.27 ^a^	7.41 ± 0.99 ^a^	9.226 ± 0.59 ^b^	2.97 ± 0.23 ^a^	3.21 ± 0.62 ^a^	3.08 ± 0.38 ^a^	27.34 ± 3.51 ^c^	32.16 ± 3.01 ^b^	27.41 ± 3.07 ^a^	0.13 ± 0.02 ^c^	0.53 ± 0.02 ^a^	0.33 ± 0.01 ^a^
KAT	2.85 ±0.29 ^a^	8.42 ± 1.21 ^a^	11.05 ± 1.09 ^a^	2.92 ± 0.19 ^a^	2.94 ± 0.48 ^a^	2.76 ± 0.22 ^a^	28.64 ± 3.99 ^c^	27.34 ± 1.78 ^c^	30.52 ± 2.79 ^a^	0.45 ± 0.05 ^c^	2.13 ± 0.22 ^a^	0.38 ± 0.04 ^a^
P9	1.07 ± 0.21 ^a^	1.54 ± 1.13 ^c^	7.39 ± 0.91 ^c^	2.47 ± 0.44 ^a^	2.85 ± 0.76 ^a^	3.02 ± 0.49 ^a^	35.68 ± 4.32 ^b^	30.22 ± 1.62 ^a^	26.14 ± 3.17 ^a^	1.68 ± 0.03 ^a^	1.15 ± 0.10 ^a^	0.4 ± 0.03 ^a^
Mean of each pigeon pea genotype for biochemical traits under different water regimes												
	APX			TPC			TSS			Total protein content		
Genotype	FC	50% FC	25% FC	FC	50% FC	25% FC	FC	50% FC	25% FC	FC	50% FC	25% FC
P1	6.37 ± 1.9 ^c^	8.39 ± 2.22 ^c^	8.87 ± 1.18 ^b^	93.34 ±4.44 ^a^	24.08 ± 0.98 ^a^	23.06 ± 1.57 ^b^	0.10 ± 0.02 ^a^	0.10 ± 0.11 ^a^	0.12 ± 0.01 ^a^	109.61 ± 11.56 ^a^	9.83 ± 2.17 ^c^	9.03 ± 1.41 ^c^
P2	85.12 ± 4.32 ^a^	20.18 ± 2.87 ^b^	5.42 ± 1.02 ^c^	33.72 ± 1.23 ^b^	20.71 ± 0.88 ^b^	38.86 ± 1.98 ^a^	0.25 ± 0.00 ^a^	0.128 ± 0.11 ^a^	0.08 ± 0.00 ^a^	19.21 ± 1.53 ^c^	14.49 ± 2.31 ^b^	16.44 ± 1.66 ^c^
P3	4.17 ± 1.47 ^c^	9.88 ± 2.01 ^c^	5.65 ± 1.07 ^c^	30.01 ± 1.14 ^b^	23.71 ± 0.76 ^a^	17.00 ± 1.01 ^c^	0.11 ± 0.01 ^a^	0.07 ± 0.11 ^a^	0.15 ±0.02 ^a^	21.27 ± 1.98 ^c^	19.51 ± 2.33 ^b^	9.99 ± 1.21 ^c^
SM	6.01 ± 1.24 ^a^	19.7 ± 2.71 ^b^	8.69 ± 1.12 ^b^	25.87 ± 1.57 ^b^	21.63 ± 1.08 ^a^	18.14 ± 1.22 ^c^	0.14 ± 0.00 ^a^	0.09 ± 0.11 ^a^	0.13 ± 0.01 ^a^	45.46 ± 3.44 ^b^	23.15 ± 2.47 ^a^	6.62 ± 0.94 ^c^
MM	1.79 ± 0.95 ^c^	12.8 ± 0.98 ^b^	8.1 ± 1.11 ^b^	24.6 ± 1.04 ^b^	17.27 ± 0.48 ^b^	18.02 ± 1.99 ^c^	0.13 ± 0.00 ^a^	0.08 ± 0.11 ^a^	0.08 ± 0.00 ^a^	51.62 ± 4.21 ^b^	9.17 ± 1.23 ^c^	18.8 ± 1.09 ^c^
LM	67.08 ± 3.77 ^b^	27.08 ± 2.43 ^a^	10.3 ± 1.32 ^a^	27.6 ± 1.84 ^b^	19.72 ± 0.53 ^b^	18.82 ± 0.98 ^c^	0.09 ± 0.00 ^a^	0.15 ± 0.11 ^a^	0.22 ± 0.02 ^a^	3.11 ± 0.98 ^c^	7.96 ± 0.99 ^c^	13.55 ± 1.01 ^c^
KAT	4.29 ± 0.88 ^c^	3.39 ± 0.97 ^c^	14.88 ± 1.01 ^a^	25.65 ± 2.33 ^b^	17.65 ± 0.79 ^b^	18.39 ± 1.14 ^c^	0.18 ± 0.01 ^a^	0.10 ± 0.11 ^a^	0.08 ± 0.01 ^a^	30.13 ± 1.89 ^c^	7.34 ± 1.67 ^c^	67.06 ± 3.032 ^a^
P9	2.98 ± 0.18 ^c^	6.19 ± 1.87 ^c^	13.21 ± 1.47 ^a^	19.93 ± 1.94 ^c^	17.31 ± 0.58 ^b^	18.87 ± 1.91 ^c^	0.23 ± 0.03 ^a^	0.15 ± 0.11 ^a^	0.12 ± 0.02 ^a^	23.06 ± 1.48 ^c^	32.56 ± 3.22 ^a^	31.11 ± 3.04 ^b^
Mean of each pigeon pea genotype for biochemical traits under different water regime												
	TAC			TFAAs				Proline				
Genotype	FC	50% FC	25% FC	FC	50% FC		25% FC	FC		50% FC	25% FC	
P1	0.04 ± 0.01 ^a^	0.04 ± 0.01 ^a^	0.16 ± 0.05 ^b^	0.44 ± 0.02 ^a^	0.33 ± 0.01 ^b^		0.60 ± 0.03 ^b^	24.44 ± 2.22 ^b^		31.62 ± 4.44 ^c^	138.52 ± 5.74 ^a^	
P2	0.03 ± 0.01 ^a^	0.07 ± 0.03 ^a^	0.08 ± 0.01 ^b^	0.31 ± 0.01 ^a^	0.59 ± 0.02 ^a^		0.67 ± 0.02 ^b^	50.16 ± 3.43 ^a^		47.6 ± 4.30 ^b^	188.49 ± 9.34 ^a^	
P3	0.12 ± 0.02 ^a^	0.06 ± 0.02 ^a^	0.04 ± 0.00 ^a^	0.17 ± 0.01 ^a^	0.37 ± 0.01 ^b^		0.81 ± 0.03 ^a^	17.95 ± 3.87 ^c^		61.94 ± 3.93 ^a^	35.07 ± 4.27 ^c^	
SM	0.02 ± 0.03 ^a^	0.38 ± 0.01 ^a^	0.64 ± 0.02 ^a^	0.04 ± 0.00 ^a^	0.04 ± 0.00 ^c^		0.64 ± 0.01 ^b^	38.28± 4.01 ^b^		52.96 ± 3.97 ^a^	154.51 ± 5.34 ^a^	
MM	0.01 ± 0.01 ^a^	0.02 ± 0.00 ^a^	0.18 ± 0.03 ^b^	0.59 ± 0.02 ^a^	0.67 ± 0.04 ^a^		0.70 ± 0.02 ^a^	21.26 ± 4.66 ^c^		26.16 ± 4.01 ^c^	124.93 ± 8.77 ^a^	
LM	0.01 ± 0.01 ^a^	0.1 ± 0.02 ^a^	0.17 ± 0.01 ^b^	0.24 ± 0.01 ^a^	0.54 ± 0.01 ^a^		0.17 ± 0.00 ^c^	21.23 ± 4.75 ^c^		22.05 ± 3.22 ^c^	149.11 ± 7.04 ^a^	
KAT	0.05 ± 0.00 ^a^	0.06 ± 0.01 ^a^	0.11 ± 0.02 ^b^	0.36 ± 0.03 ^a^	0.54 ± 0.02 ^a^		0.55 ± 0.01 ^b^	18.96 ± 4.01 ^c^		39.06 ± 3.55 ^b^	137.13 ± 6.97 ^b^	
P9	0.03 ± 0.01 ^a^	0.05 ± 0.02 ^a^	0.07 ± 0.00 ^b^	0.22 ± 0.01 ^a^	0.13 ± 0.00 ^c^		0.43 ± 0.02 ^b^	15.89 ± 3.92 ^c^		36.2 ± 3.63 ^b^	111.48 ± 8.68 ^b^	

Traits: TPC—Total Phenolic Content; MDA—Malondialdehyde content; TSS—Soluble sugars; TAC—Total amino acids; TFAA—Total Free Amino Acids; H_2_O_2_—Hydrogen peroxide; POD—Total Peroxidase; CAT—Catalase; APX—Ascorbate Peroxidase. In the same column, different lowercase letters represent significant alterations among pigeon pea within the same stress level, according to Scott–Knott’s test (*p* < 0.05).

The catalase (CAT) activity varied among the pigeon pea genotypes under different water treatments. In an inter-genotypic comparison under various drought stress treatments, genotype KAT exhibited the highest CAT activity under moderate drought stress (2.13 U/mg protein), followed by genotypes P1 (1.58 U/mg protein), SM (1.25 U/mg protein), P9 (1.15 U/mg protein), MM (0.68 U/mg protein), P3 (0.58 U/mg protein), LM (0.53 U/mg protein), and P2 (0.45 U/mg protein) (Table 3). There was also an increase in catalase activity under severe drought stress treatments in genotypes SM (0.93 U/mg protein), P3 (0.85 U/mg protein), MM (0.78 U/mg protein), and LM (0.33 U/mg protein) compared to their respective control treatments. Conversely, genotypes P1, KAT, and P9 registered a decrease in catalase activity under severe drought stress treatment compared to their respective control treatments. Genotype P2 registered similar CAT activity under severe drought stress as its control treatment (0.33 U/mg protein) (Table 3).

There was a significant (*p* ≤ 0.05) increase in ascorbate peroxidase (APX) activity observed in genotypes P1, P3, SM, MM, and P9 under moderate drought stress compared to their corresponding control treatments. However, both genotype P2 and LM exhibited a significant decline in APX activity as the severity of drought intensified (Table 3). Genotype KAT displayed a decrease in APX activity under moderate drought stress but experienced a significant increase under severe drought stress (Table 3).

### 2.6. Effect of Water Stress on Phenolic Acids

The total phenolic content in the pigeon pea genotypes varied at different water availabilities (Table 3). At field capacity (FC), genotypes P1 and P9 had the highest (93.34 mg/g FW), and lowest (19.93 mg/g FW) concentrations, respectively. At 50% FC, most genotypes showed a decrease in phenolic acids, with genotypes P1 and MM having the highest (24.08 mg/g FW) and lowest (17.27 mg/g FW), respectively. At 25% FC, total phenolic content declined further, with genotypes P2 and P3 having the highest (38.86 mg/g FW) and lowest (17.00 mg/g FW), respectively (Table 3).

### 2.7. Effect of Water Stress on Osmolytes

#### 2.7.1. Total Soluble Sugars

There were no significant (*p* > 0.05) change in total soluble sugars among the pigeon pea genotypes under moderate and severe drought–stressed conditions. Genotype P1 exhibited a slight increase in total soluble sugar (TSS) accumulation under severe drought stress. However, TSS for both control and moderate stress conditions remained the same. Additionally, a decline in TSS was observed in genotype P2 as drought severity increased compared to the controls. Genotype P3 showed a decrease in TSS under moderate drought stress but registered an increase in TSS under severe drought stress. Genotype SM experienced a slight decline in TSS under both moderate and severe drought stress compared to its control treatment. The MM genotype showed a slight decline in TSS under both moderate and severe drought stress treatments. The LM genotype displayed a steady increase in TSS accumulation with increasing drought stress intensity. Genotype KAT exhibited a decrease in TSS accumulation under moderate drought stress and a further decrease in TSS under severe drought stress treatment. Genotype P9 showed a steady decrease in TSS accumulation as drought severity increased (Table 3).

#### 2.7.2. Proteins and Free Amino Acids

Under 100% FC conditions, the total protein content varied among the pigeon pea genotypes, with P1 having the highest at 109.61 mg/mL and the LM genotype the lowest at 3.11 mg/mL (Table 3). As an observed trend, the total protein content decreased in genotypes P1, P2, P3, SM, and MM as drought severity increased. However, genotypes LM, KAT, and P9 exhibited an increase in total protein content as drought severity increased compared to their respective controls.

The total amino acid content in the pigeon pea genotypes varied across different water stress treatments (Table 3). At field capacity (FC), the content ranged from 0.01 mg/g FW (for LM genotype) to 0.12 mg/gFW (for genotype P3). Under moderate water stress (50% FC), some genotypes increased their total amino acid content, with genotype SM having the highest at 0.38 mg/gFW. Under severe drought stress (25% FC), most genotypes exhibited increased content, with genotype SM having the highest at 0.639 mg/gFW.

Total free amino acids (TFA) concentrations in the pigeon pea genotypes exhibited notable variations (Table 3). At FC, TFA concentration ranged from 0.04 mg/gFW (genotype SM) to 0.44 mg/gFW (genotype P1). As drought stress intensified from normal conditions to extreme drought stress, the TFA increased in genotypes P1, P2, P3, SM, MM, KAT, and P9. However, genotype LM exhibited a decline in TFA under extreme drought stress while having a higher concentration of TFA under moderate drought stress compared to its control treatment.

#### 2.7.3. Proline

The proline concentration in the pigeon pea genotypes showed variations under different water availability conditions (Table 3). At field capacity (FC), the highest (50.16 µg/mL) and lowest (15.89 µg/mL) proline concentrations were observed in genotypes P2 and P9, respectively. Under moderate water stress (50% FC), there was a significant increase, with genotype P3 displaying the highest at 61.94 µg/mL. Under severe water stress (25% FC), proline concentration further increased, with genotype P2 registering the highest at 188.49 µg/mL.

### 2.8. Genetic Variation Among Pigeon Pea Genotypes Using SCoT Markers

The use of 20 SCoT markers resulted in the successful amplification of a total of 848 distinct and clear bands, spanning a size range of 150 to 3000 base pairs. The average count of bands per primer was 42 across the eight genotypes varying from a minimum of 4 bands for SCoT29 to a maximum of 96 bands for SCoT23. Representative agarose gel images of the amplicons are represented in Supplementary Figure S1.

In evaluating the 20 SCoT markers, polymorphic information content (PIC) values were determined, yielding an average of 0.34. The lowest PIC score (0) was observed for SCoT10, while the highest value (0.38) was recorded for SCoT7. Moreover, the mean resolving power (Rp) was established at 4.79. The range of RP values spanned from a minimum of 0 for SCoT 10 to a maximum of 11 for SCoT3. The mean heterozygosity (He) assessment at the cultivar level across the 20 SCoT markers was computed as 0.44. SCoT10 exhibited a minimum value of 0, and a cluster of markers (SCoT3, 6, 7, 8, 14, 15, 21, 29, 30, and 32) shared the maximum value of 0.5 (Table 3).

This study also revealed that 64.92% of amplified bands exhibited polymorphism. SCoT2 and SCoT35 markers demonstrated 100% polymorphic profile across the eight evaluated pigeon pea genotypes. SCoT10 marker exhibited no polymorphism (Table 4). At the genotypic level, the average score for gene diversity (He) was 0.491. The genetic diversity score ranged from 0.468 for genotype P3 to 0.500 for genotypes P2, LM, and KAT (Supplementary Table S3).

#### 2.8.1. Clustering of Pigeon Pea Genotypes

**Figure 1:**
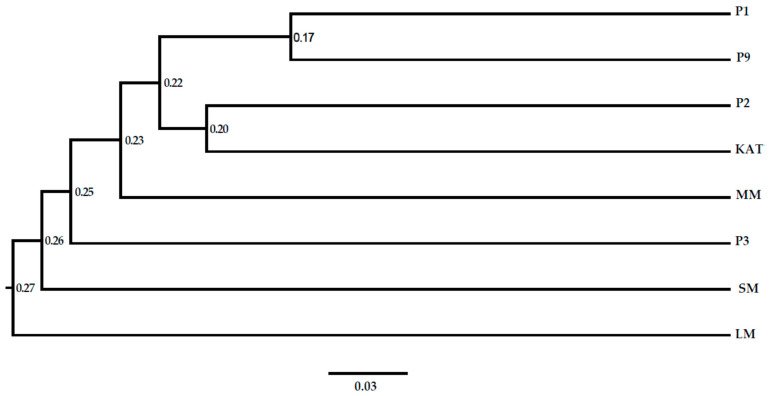
Unweighted pair group method of arithmetic mean (UPGMA) dendrogram based on Jaccard’s coefficient pigeon pea genotypes using SCoT markers. Genotypes: P1, P2, P3, SM, MM, LM, KAT, and P9.

Unweighted pair group method of arithmetic mean (UPGMA) cluster analysis of SCoT marker data grouped the eight genotypes into two main clusters (cluster 1 and 2) at a genetic similarity coefficient of 0.95 (Figure 1). Cluster 1 was composed of genotypes P1, P2, P9, and KAT collected from Machakos and Kitui Counties. Cluster 2 comprised of the remaining four (MM, P3, SM, and LM) from Genebank of Kenya and Kitui County. It shows that the genotypes did not group according to geographical regions. The LM genotype is closely grouped with the SM genotype and P3, suggesting a strong genetic connection between them. Additionally, genotypes P1 and P9 are closely grouped, showing a close genetic relationship. Similarly, genotypes P2 and KAT are also closely grouped, highlighting their close relationship. The MM genotype shares two ancestors with P1, P9, P2, and KAT as demonstrated by two branching points in the dendrogram. This suggests a complex genetic relationship with these other genotypes.

#### 2.8.2. Similarity Coefficient Among the Eight Pigeon Pea Genotypes

Jaccard’s coefficients exhibited a range extending from 0.336 to 0.676. The computed average similarity index was 0.607. The most dissimilar pair was observed between genotypes P1 and P9, exhibiting a similarity index of 0.336. Conversely, the highest similarity was between genotypes SM and MM, with a similarity index of 0.676 (Table 5).

#### 2.8.3. Principal Coordinate Analysis (PCoA)

**Figure 2:**
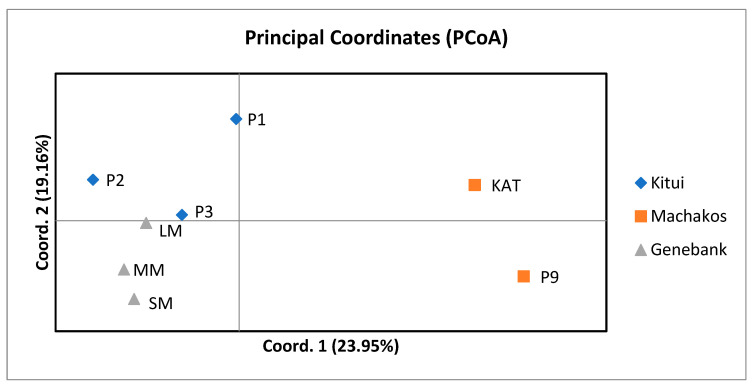
Principal coordinate analysis (PCoA) of the eight pigeon pea genotypes collected from Genebank of Kenya (Kitui and Machakos Counties) as revealed by the 20 SCoT markers.

Principal coordinate analysis (PCoA) classified the eight pigeon pea genotypes into two primary clusters, alongside two individual outgroups (Figure 2). The initial two components of the principal coordinates collectively accounted for 43.11% (23.95% and 19.16% for components 1 and 2, respectively) of the overall variation. In the first quadrant, genotypes P1, P2, and P3, collected from Kitui, exhibited a close clustering pattern. In the third quadrant, genotypes from the Genebank, including SM, MM, and LM demonstrated a closely linked association. The genotypes from Machakos formed clusters in both the second and fourth quadrants of the principal coordinate analysis.

Analysis of molecular variance (AMOVA) indicated a substantial level of variation within populations (*p* < 0.001), while no significant variation was observed among populations (Table 6). The variation within population accounted for 80% of the overall variation exhibited by the accessed genotypes.

## 3. Discussion

Drought stress poses a significant challenge to crop productivity and pigeon pea (*Cajanus cajan* L.) is no exception to its impact. The present study confirmed the significant reduction in shoot and root growth under drought stress. The shoot and root inhibition pattern and reduction in biomass production also differed among the four genotypes when subjected to drought stress. The differential genotype response to drought stress suggests a great deal of genetic variation among genotypes. The growth suppression and biomass reduction were lowest in LM, KT, and SM compared to other pigeon pea genotypes in drought stress conditions, indicating its ability to withstand drought stress. Therefore, these parameters could be used as morphological criteria for detecting drought stress tolerance and susceptibility in pigeon pea plants.

In the present study, under escalating water stress, pigeon pea plants exhibited a substantial decrease in the quantum yield of photosystem II (Phi 2) across all the eight genotypes, aligning with findings previously reported in *Lablab purpureus* [44]. This reduction demonstrates the photosynthetic sensitivity of pigeon pea to water deficit, with diverse responses highlighting the effect of genotypic variations. The photosystem II quantum efficiency (FvP/FmP) ratio, a crucial indicator of photosystem II (PSII) photochemical efficiency, consistently declined with the severity of water stress, providing a direct measure of plant stress exposure [45]. Despite initial higher values under moderate water stress, the FvP/FmP ratios significantly reduced under extreme stress conditions, consistent with trends observed in soybeans due to increased susceptibility to photo-oxidative stress, and impaired PSII efficiency [46,47]. Under water stress conditions, all the pigeon pea genotypes exhibited a consistent decline in photochemical efficiency (FvP/FmP) coupled with a notable increase in non-photochemical quenching (NPQt). This heightened NPQt acts as a protective mechanism for PSII against damaging conditions, dissipating excess excitation energy as heat [46,48]. The dynamic response of NPQt under different stress levels, especially its dramatic increase under severe water stress, highlights the plant’s effective regulation of excess energy, preventing potential photodamage [49,50]. Additionally, the positive correlation between Phi2 and FvP/FmP suggests that genotypes with higher PSII photochemistry efficiency also enhance overall photosynthetic efficiency, potentially impacting improved crop yields [51,52].

This study revealed that the genotypes LM and KAT consistently maintained RWC levels and photosynthetic pigments, which acted to maintain high photosynthetic efficiency in these genotypes under water stress conditions. This is corroborated by the unaltered levels of soluble sugars in the leaves of drought-stressed plants. This aligns with previous studies demonstrating that genotypes exhibiting superior water retention capabilities are inherently more resilient to drought stress [53,54]. The findings from this study confirms earlier studies [55], emphasizing the adverse impact of water stress on the RWC in plants. The observed variability in the RWC in the pigeon pea genotypes in response to water stress demonstrates the genetic variability in drought tolerance [54]. The genotypes LM and KAT characterized by their ability to maintain higher RWC levels and photosynthetic pigments emerged as potential drought-tolerant candidates. Proline accumulation in severely stressed plants of genotypes LM and KAT might play an active role in the regulation of the RWC and membrane damage from growth recovery as previously reported for cotton [56], rice [57], *Pinus ponderosa* trees [58], and wheat [59,60] under water stress.

Previous studies have reported increased ROS production in response to drought stress across diverse plant species [18,61,62,63]. In this study, a significant increase in MDA content and H_2_O_2_ occurred in the leaf tissues of all eight pigeon pea genotypes under severe water stress conditions. The elevated levels of these oxidative stress markers contribute to the destruction of photosynthetic pigments, protein denaturation, and eventually programmed cell death [64,65]. Water-stressed pigeon pea genotypes displayed elevated activities of ascorbate peroxidase (APX) and catalase (CAT) compared to control plants, indicating enhanced plant tolerance to oxidative damage by reinforcing antioxidant defence mechanisms [66]. Notably, leaves of drought-stressed plants exhibited a significant rise in CAT activity, reaching levels of 2.13 units/mg protein in genotype KAT. This may be attributed to the increased accumulation of H_2_O_2_ and MDA, leading to the excitation of the activities of ROS scavenging enzymes. Fluctuations in catalase activity among genotypes could be as a result of unique molecular responses to water deficit, reflecting genetic heterogeneity in drought stress responses in diverse genotypes [67,68]. Some genotypes, including P1, P3, SM, and MM, showed increased catalase activity under water stress, suggesting their high capacity to upregulate enzyme production to protect against ROS accumulation and oxidative damage.

Peroxidases, essential for detoxifying ROS, play a crucial role in safeguarding plant cells from oxidative damage [69,70]. Genotypes P1, P3, P9, SM, MM, and KAT exhibited increased APX activity under water stress. Peroxidase activity (POD) was significantly increased under water stress for genotypes P3, SM, MM, LM, and KAT. The increased scavenging enzymes activity in these genotypes under water stress suggests their capacity to upregulate enzyme production to protect against ROS accumulation and oxidative damage. The decline in enzymatic activities in some genotypes indicates that these genotypes might have a low capacity to catalyse reactive oxygen species under water stress conditions. This may also suggest that these genotypes may be susceptible to prolonged drought stress and oxidative damage [71,72,73].

Phenolic compounds as secondary metabolites play a vital role in plants’ adaptation and defence mechanisms under adverse environmental conditions, including drought stress. The reduction in total phenolic compounds (TPCs) across these genotypes suggests potential consequences in their ability to withstand and adapt to drought stress. However, a notable exception to this trend was observed in genotype P2, which showed an increase in the accumulation of total phenolics compared to its control. The observed increase in phenolic content in genotype P2 under water stress conditions may be due to enzyme activation [74]. This suggests that genotype P2’s response to drought could be regulated by phenolic acids, offering a potential approach to improve drought tolerance in pigeon pea through manipulation of the phenylpropanoids pathway [75,76,77].

The increased sensitivity to drought in the pigeon pea genotypes is linked to a decrease in soluble carbohydrate accumulation, likely a consequence of impaired CO_2_ assimilation under water limitation [78,79]. The higher concentration of total soluble sugars in control plants, compared to drought-stressed plants, is attributed to the abundance of water, facilitating efficient sugar synthesis and accumulation through photosynthesis [80]. Water stress reduced the total soluble sugar content in five pigeon pea genotypes (P2, P9, SM, MM, and KAT) accompanied by significant decreases in Phi2, FvP/FmP, and SPAD values. This decline in FvP/FmP, indicating PSII quantum efficiency, suggests a potential decrease in photosynthesis, leading to reduced sugar synthesis. Conversely, drought stress increased the total sugar content in genotypes P1, P3, and LM, despite declines in photosynthetic parameters Phi2, FvP/FmP, and SPAD. This increase may result from reduced plant growth before photosynthesis under moderate drought [81], leading to an excess of carbon skeletons redirected towards osmolyte production [82]. Under adverse conditions, the soluble sugar is essential for maintaining membrane integrity and adjusting osmotic pressure.

The protein content analysis revealed an increase in three out of the eight pigeon pea cultivars—specifically, genotypes P9, LM, and KAT. This elevation in protein levels under water stress aligns with the notion that stress-induced proteins may serve as a form of nitrogen storage, utilized later by the plant, or contribute to osmotic adaptation [83,84]. Drought stress can intricately influence protein synthesis, with evidence suggesting both the induction of new proteins to enhance plant survival and, paradoxically, the degradation of leaf proteins under severe water deficiency, leading to a shift towards free amino acids and decreased protein synthesis [85,86,87]. This study revealed a significant increase in total free amino acids under intensified drought stress across the pigeon pea genotypes, except for genotype LM, which showed a decline under severe water stress. Notably, genotype P3 exhibited the most substantial rise in free amino acid content, highlighting its pivotal role as an adaptation strategy during water deficit stress conditions. These amino acids contribute to detoxifying reactive oxygen species, regulating pH, and facilitating osmotic adjustments [88,89]. This increase in free amino acids is attributed to heightened protein degradation, which enhances osmotic potential and drought tolerance, while also serving as a reservoir of nitrogen and carbon for essential metabolic processes.

The results reveal a linear increase in proline content with escalating drought stress, with genotypes P2, SM, and LM exhibiting the highest proline accumulation under severe water stress conditions. This increased proline accumulation aligns with its established role as an osmo-protectant and stress-responsive molecule, crucial for maintaining osmotic balance, stabilizing cellular structures, and mitigating the negative impacts of drought stress [90,91,92]. The significance of proline in drought tolerance lies in its contribution to cellular protection against osmotic stress, stabilization of macromolecules, and maintenance of redox balance, as well as serving as a reservoir of carbon and nitrogen for future metabolic demands after stress alleviation. The increase in proline levels in pigeon pea plants experiencing severe water stress might actively contribute to maintaining the relative water content (RWC) and mitigating membrane damage [93]. Despite this consistent pattern, genotype P3 exhibited a sustained low-level increase in proline content under extreme water stress. These diverse responses underscore the genetic variability in stress tolerance mechanisms among the pigeon pea genotypes, with genotype P2 showcasing a robust potential for proline accumulation as part of their stress adaptation strategy.

The observed variability in proline accumulation within the pigeon pea genotypes resonates with similar findings in rice and wheat, emphasizing the genetic variability underpinning stress responses [94,95]. This diversity carries implications for breeding strategies aimed at enhancing drought tolerance in pigeon pea, urging the prioritization of genotypes with a higher capacity for proline accumulation. The significance of proline in drought response is further supported by studies in other plant species such as strawberries, peas, and petunias, where proline accumulation and antioxidant enzyme activity were upregulated in response to drought stress [96,97,98]. These consistent observations across diverse plant species underscore the conserved nature of the role and regulation of proline in response to water deficit [91], reinforcing its importance in the context of drought stress adaptation in pigeon pea plants.

Understanding genetic diversity within pigeon pea populations is critical for guiding future breeding initiatives, ensuring the preservation of necessary genetic diversity for a robust breeding program [99]. The current study revealed low levels of observed heterozygosity (0.40) and gene diversity (GD) (0.44) among the studied population, indicating a prevalence of homozygous individuals sharing common alleles. The reduced heterozygosity aligns with the self-pollinating nature of *C. cajan* [100] and is consistent with findings among Tanzanian pigeon pea accessions [101]. The polymorphic information content (PIC) values ranged from 0.0 to 0.39, averaging 0.335, indicating moderate genetic variability, consistent with findings in other legume crops [99,102,103]. The observed variation in PIC values can be attributed to genotypic differences within the pigeon pea genotypes, with one marker (SCoT10) showing a PIC value of 0, thus indicating its inability to discriminate between genotypes. The moderate PIC value in this study indicates a moderate degree of polymorphism among the pigeon pea genotypes, facilitating a precise estimation of genetic distance. Among the primers, SCoT7 and SCoT29 displayed the highest PIC values (0.38), suggesting their moderate utility for assessing genetic diversity within the pigeon pea genotypes.

The principal coordinate analysis (PCoA) results revealed three distinct sub-populations influenced by geographical adaptation. The pigeon pea population from Kitui County showed the highest genetic similarity, emphasizing the importance of considering geographical origins in breeding decisions. To validate differentiation between populations, the findings indicated significant molecular variance among and within populations. The distribution of variance within populations aligns with observations in other cross-pollinating species and studies involving SCoT markers, contrasting with self-pollinating plant species, which typically exhibit greater genetic diversity among populations [27].

The analysis of molecular variance uncovered a significant proportion of genetic variation within populations, contrasting with a relatively low difference among the three studied populations. These findings are similar to the observations by Kimaro et al. [101], who reported a higher genetic variation of 53.3% within pigeon pea accessions using SSR markers. The restricted genetic diversity within pigeon pea cultivars, encompassing only a fraction of the overall genetic diversity present in the pigeon pea gene pool, aligns with previous research [104,105]. This study reinforces earlier findings indicating limited genetic variation among the cultivated pigeon pea genotypes collected in Africa [106].

The SCoT markers demonstrated low genetic diversity among the eight tested pigeon pea genotypes. The observed low genetic diversity can be attributed partly to the self-pollinating nature of pigeon pea and the limited geographic origins of the studied genotypes [107,108], which were obtained from the Eastern Province region of Kenya. The implications of limited genetic diversity within these genotypes are particularly significant for pigeon pea breeding programs since the adoption of genetically homogeneous cultivars has historically led to reduced plant genetic diversity, rendering crops susceptible to diseases and pests [106]. Pigeon pea, despite its morphological diversity, lacks comparable molecular-level diversity, emphasizing the need for strategic breeding decisions.

## 4. Materials and Methods

### 4.1. Plant Materials and Effect of Drought on Pigeon Pea Accessions at the Seedling Stage

Eight pigeon pea landraces were used in the experiment, three of which were obtained from the National Genebank of Kenya domiciled at the Genetic Resources Research Institute (GeRRI) of the Kenya Agricultural and Livestock Research Organisation (KALRO) and five from farmers’ fields in Lower Eastern (Machakos and Kitui Counties), Kenya.

The experiment was carried out using a completely randomized design with five replications. Three seeds of each pigeon pea landrace were grown in pre-weighed soil in 15 cm diameter plastic pots and left to germinate under greenhouse conditions. Thinning was performed on the seventh day and only one seedling was left to grow in each pot. On the 14th day, three stress treatments were induced by adjusting water content to 100% field capacity (FC) as a control, 50% FC (moderate drought stress), and 25% FC (severe drought stress). Drought stress was applied by first subjecting all the plants to maximum saturation level for 24 h after which, the water was emptied from the holding trays. The water absorbed was allowed to drain for 24 h for the soil to attain field capacity (FC) retention. Subsequently, the weights of individual pots were taken and the amount of water retained in the soil was recorded. Drought stress was induced by maintaining the pots with 50% FC and 25% FC. The pots were monitored daily and watered to their respective weights. The amount of water added to each pot was calculated using the percentage of pot water capacity [109].

### 4.2. Assessment of Growth and Biomass Production Parameters

Five randomly selected pigeon pea plants were taken to evaluate the growth performance by measuring the shoot length (SL) and the root length (RL) in plants grown under normal and drought stress conditions. The data were collected after exposure to drought stress treatments for 28 days. Plants along with the roots were carefully removed from the soil, the roots were rinsed gently with tap water, and were then placed in tissue paper to absorb moisture. The fresh weights (FW) of individual plants were measured by using an electronic weighing machine (ELICO Electronic, Georgiev KD, Bulgaria). Afterwards, the plants were wrapped in aluminium foil and kept in an oven at 70 °C for 72 h to obtain the dry weight (DW).

### 4.3. Measurement of Physiological Parameters

After exposure to drought stress treatment for 28 days, measurements of physiological variables including photosynthetic rate, chlorophyll content (SPAD), leaf temperature (LT), linear electron flow (LEF), quantum yield of photosystem II (Phi2), ratio of incoming light (excited electrons) that goes towards non-photochemical quenching (PhiNPQ), ratio of incoming light that is lost via non-regulated processes (PhiNO), non-photochemical quenching (NPQt), and maximum quantum yield of photosystem II (FvP/FmP) were analysed using MultspeQ Beta Device v1.0 (East Lansing, MI, USA). In addition, the leaf samples were harvested and used to determine the relative water content (RWC).

### 4.4. Determination of the Relative Water Content

The estimation of the relative water content was determined according to the protocol described by Mullan and Pietragalla [110]. Fully expanded leaves that were exposed to maximum sunlight were selected from the plants in the glasshouse on the 14th day after the start of the drought stress treatment. The leaf samples were placed in pre-weighed sample tubes and hermetically sealed with a lid. Subsequently, the individual tubes were weighed to obtain the fresh weight (FW) of the leaves. Distilled water (1 mL) was added to each tube, and the tubes were then kept in darkness at 4 °C for 24 h to enable the leaves to attain full turgor. The leaf samples were then carefully removed from the tubes and blotted dry with paper towels. The turgid weight (TW) was measured by weighing each leaf sample. The leaf samples were then placed in labelled envelopes and dried for 48 h in an oven at 70 °C. The dry weight was recorded, and the relative water content (RWC) was calculated using the formula

$$WC=\frac{\left(FW-DW\right)}{\left(TW-DW\right)}\times 100$$

where FW is the fresh weight, DW is the dry weight, and TW is the turgid weight.

### 4.5. Determination of the Malondialdehyde and Hydrogen Peroxide Content

The content of MDA was determined as described by Shin et al. [111], whereby leaf tissue (0.5 g) of stressed and non-stressed plants were ground into a fine paste using 2 mL of ice-cold trichloroacetic acid (0.1% *w*/*v*). The fine homogeneous paste was centrifuged at 18,626× *g* for 10 min at 4 °C. Exactly 0.5 mL of the supernatant was mixed with 1.5 mL solution of 10% trichloroacetic acid and 0.25% thiobarbituric acid (TBA) reagent. The mixture was then placed in a hot water bath at 95 °C for 30 min, cooled on ice for 5 min, and centrifuged at 855.3× *g* for 10 min. To quantify the MDA concentration, 200 μL of the supernatant was subjected to spectrophotometric analysis by measuring the absorbance (A_532_ and A_600_). To the supernatant, 1 mL of 20% (*w*/*v*) TCA containing 0.5% (*w*/*v*) TBA was added and incubated in a water bath at 95 °C for 30 min and quickly cooled on ice. The mixture was spun at 9503× *g* for 10 min and the absorbance was measured at 532 and 600 nm using a UV-mini 1240 spectrophotometer (Shimadzu, Kyoto, Japan). A molar absorptivity value of 155 mM^−1^ cm^−1^ was deployed to calculate the MDA content after taking away the non-specific turbidity reading at 600 nm. The malondialdehyde concentration was calculated with its extinction coefficient 155 mM^−1^ cm^−1^ and expressed as nmol malondialdehyde g^−1^ fresh mass using the formula

$$MDA\;content=Extaction\;buffer\left(mL\right)\times Supernatant\left(mL\right)[\frac{A532-A600}{155}\times \frac{1000}{Amount\;of\;Sample}]$$

where A532 nm denotes the maximum absorbance of the TBA-MDA complex, A600 nm represents the correction for non-specific turbidity, and 155 mM^−1^ cm^−1^ is the specific molar extinction coefficient for MDA.

All measurements were conducted three times using three independent extracts from one plant sample.

The hydrogen peroxide content was measured as described by Velikova et al. [112]. Briefly, 0.5 g leaf sample was homogenized with 2 mL cold 0.1% (*w*/*v*) TCA. The suspension was spun at 1164× *g* for 30 min at 4 °C. Then, 0.5 mL of the supernatant, 0.5 mL 10 mM potassium phosphate buffer (pH 7.0), and 1 mL of 1 M potassium iodide was added and mixed. The absorbance was read at 390 nm and the amount of H_2_O_2_ was calculated using the extinction coefficient 0.28/mM/cm and expressed as μmol/g FW (fresh weight).

### 4.6. Determination of Antioxidant Enzyme Activities

Leaf samples from water-stressed plants were collected 14 days after the initiation of drought stress. These samples were then crushed into a fine paste using a sterilized cold mortar and pestle in 2 mL of extraction buffer (100 mM KHPO_4_ (pH 6.8), 0.2 mM EDTA, and 1% (*w*/*v*) polyvinylpyrrolidone). The mixture was then spun in a Mikro 200R centrifuge (Andreas Hettich Gmbh, Tuttlingen, Germany) at 19,980× *g* for 20 min at 4 °C. The supernatants were then assayed for ascorbate peroxidase, total peroxidase, and catalase enzyme activities.

Ascorbate peroxidase (APX) activity was assayed by monitoring ascorbic acid oxidation and recording the change in absorbance at 290 nm. The leaf extract (10 µL) was mixed with 2 mL reaction buffer (0.2 mM Tris/HCl buffer (pH 7.8), 0.5 mM hydrogen peroxide, and 0.25 mM ascorbic acid). The activity of APX was calculated from the extinction coefficient (2.8 mM^−1^ cm^−1^) of ascorbate as delineated by Nakano and Asada [113]. The APX activity was expressed in units per mg of protein (U/mg protein).

Total peroxidase (POD) activity was assayed by adding 50 µL of the leaf extract to 2 mL reaction mixture (0.05 M sodium acetate buffer (pH 7.0), 0.025 M guaiacol, and 0.025 M hydrogen peroxide). The total peroxidase activity was determined by recording the increase in absorbance due to the formation of tetra-guaiacol at 470 nm, taking the coefficient of extinction for the reaction to be 26.6 mM^−1^ cm^−1^. The POD activity was expressed in units per mg of protein (U/mg protein).

The catalase (CAT) activity was determined following the Cakmak et al. [114] protocol. First, 50 μL of the enzyme extract, 3 mL of reaction buffer (15 mM H_2_O_2_ and 50 mM phosphate buffer (pH 7.0)) was added. Then, catalase activity was determined from absorbance readings at 240 nm for 1 min; the readings decreased with the decay of H_2_O_2_. The activity of CAT was subsequently calculated as described by Nakano and Asada [113] using the formula

$$Total\;catalase\;activity=\frac{\left(Change\;in\;absorbance\times Total\;reaction\;volume\right)}{\left(sample\;volume\times Extinction\;coefficient\;of\;the\;enzyme\right)}$$

The CAT activity was expressed in units per mg of protein (U/mg protein).

### 4.7. Determination of Total Soluble Sugars (SS)

The phenol-sulphuric acid technique as described by Chen et al. [115] was used to quantify soluble sugars. Briefly, leaf samples (0.2 g) were homogenized in 70% ethanol, and the resulting homogenates were centrifuged at 6082× *g* for 10 min. A 2 mL aliquot of the carbohydrate solution was mixed with a 5% phenol solution and concentrated sulphuric acid, followed by incubation and colour development for 10 min. Optical density readings at 490 nm were taken against dilute sulphuric acid as a blank. Redistilled phenol and freshly prepared 5% phenol in water were used. Glucose standard solution facilitated sugar quantification. The concentration of carbohydrates was calculated based on D-glucose calibration using the following equation:

Carbohydrates concentration [μg mL^−1^] = A_490_ − β/α

where A_490_ is the final absorbance at 490 nm, α represents the equation of the slope, and (β) denotes the intercept of the slope.

### 4.8. Determination of the Total Phenolic Content (TPC)

The total phenolic content in pigeon pea leaf samples was determined following the protocol of Ainsworth and Gillespie [116]. Leaf samples (0.5 g) were ground in absolute ethanol and incubated at 25 °C for 48 h. The supernatant (1 mL) obtained after centrifugation was mixed with 5 mL of 0.2 N Folin–Ciocalteau reagent (FCR) and 4 mL of sodium carbonate. After incubation for 1 h at 25 °C, optical density was measured at 765 nm, and phenolic content was quantified using a gallic acid standard curve. The results were expressed as mg of gallic acid equivalent (GAE) per gram of extracts (mg/gFW). The gallic acid standard curve was prepared using concentrations of gallic acid solutions (25, 50, 75, and 100 μg/mL) with an R^2^ value of 0.9523. The total phenolic content was expressed as mg GAE per 100 g of dry sample (mg/100 g) after correcting for dilution.

### 4.9. Estimation of Total Soluble Proteins (SP), Total Free Amino Acids (TFA), and Proline Content

The Bradford assay method [117] was used to determine the quantities of total soluble proteins in both non-stressed and drought-stressed plants. Specifically, 1 mL of the Bradford reagent was mixed with 0.1 mL of the crude extract, and the optical density was measured at 595 nm. The protein concentrations were approximated using the regression equation derived from a standard curve prepared with 1 mg/mL bovine serum albumin (BSA).

Total free amino acids in non-stressed and drought-stressed pigeon pea plants were assessed through a ninhydrin-based colorimetric assay following Huang and Wu’s protocol [118]. Leaf samples (0.2 g) were homogenized in 5 mL of absolute ethanol, and amino acids were extracted by heating the crude extracts in a water bath at 95 °C for 1 h. After cooling, the mixture was centrifuged at 9500× *g* for 10 min, and 1 mL of the supernatant was mixed with 0.5 mL of 2% (*w*/*v*) ninhydrin and 0.2 M phosphate buffer (pH 8.0). The absorbance was measured at 570 nm using a UV–Vis spectrophotometer (Shimadzu, Kyoto, Japan), and proline reference standards were prepared at a concentration of 1 mg/mL.

To quantify proline content, 0.1 mg of fresh leaf tissues from both non-stressed and drought-stressed samples were homogenized in 3% aqueous sulphosalicylic acid. After centrifugation at 9503× *g* for 5 min at room temperature, reaction mixtures were prepared with 1 mL of plant extract supernatant, 1 mL of sulphosalicylic acid, and 2 mL each of glacial acetic acid and acidic ninhydrin. After incubation at 96 °C for 1 h and termination, the reacted samples were extracted with toluene, and absorbance was measured at 520 nm using toluene as a blank. Proline concentrations were estimated based on a standard concentration curve, calculated on a fresh weight basis, and expressed as milligrams per gram FW.

### 4.10. Genomic DNA Extraction

The DNA extraction from fresh leaves of pigeon pea landraces followed the cetyltrimethyl bromide (CTAB) protocol [119]. The resulting pellet was dissolved in 50 µL sterile water and treated with RNAase (10 μg/mL) and DNA quantified spectrophotometrically (UV-mini 1240, Shimadzu-Japan). Genomic DNA size and quality were verified through agarose gel electrophoresis (1% *w*/*v* agarose gel, 0.05 mg/mL ethidium bromide) at 80 V for 60 min in an electrophoresis tank (Bio-Rad, Gmbh—FeldKirchen, Germany).

### 4.11. PCR Amplification of SCoT Markers and Product Electrophoresis

The PCR reaction of the isolated genomic DNA was carried out in a 25 μL reaction volume, which included 2.0 μL DNA (50 ng/μL), 12.5 μL of Ampliqon Taq DNA polymerase (Ampliqon A/S, Odense M, Denmark), 1.0 μL of SCoT primers (Macrogen Europe, Maastrichit, The Netherlands; Table 7), and 9.5 μL of sterile double-distilled water. The amplification was performed in a PTC-1196 thermocycler (Bio-Rad, Hercules, CA, USA) under the following conditions: pre-heating for 5 min at 94 °C; 35 cycles, with each cycle lasting 45 s at 94 °C; annealing temperature varying depending on the primers and carried out for 45 s; elongation at 72 °C for 60 s; and finally a final extension at 72 °C for 7 min. The amplified products were visualized using 1% agarose gel stained with ethidium bromide (0.05 mg/mL) and on a Gel-Doc ^TM^ XR+ Imaging System (Bio-Rad, Gmbh – FeldKirchen, Germany) under UV light. The size of the bands was estimated with reference to GeneRuler 1 kb and 100 bp ladder standard markers (Fischer Thermo Scientific, Waltham, MA, USA).

### 4.12. PCR Scoring and Data Analysis

The resulting amplicon patterns were scored as either absent (0) or present (1) following the method described by Collard and Mackill [39]. These patterns were subsequently compared to assess the genetic relationship between the eight pigeon pea genotypes.

Genetic parameters were computed using GenAlEx V6.503 software [120]. These parameters included total amplified bands (TAB), monomorphic bands (MB), percentage bands per loci (PB), resolving power (Rp), heterozygosity (HT), effective number of alleles (Ne), genetic diversity (GD), and the Shannon Information Index (I). PowerMarker v2.3.4 [121] was used to calculate the polymorphic information content (PIC) of each genotype.

### 4.13. Phylogenetic Analysis

Jaccard’s similarity coefficient was applied in determination of the pairwise relationship between markers. For cluster analysis, an unweighted pair-group method with arithmetic averages (UPGMA) dendrogram was generated, employing Nei’s genetic distance calculated from pairwise comparisons. The computation of genetic distances between pairs of accessions was carried out using GenAlEx v6.503 [120]. The distance matrix served as the basis for conducting a principal coordinate analysis (PCoA). Subsequently, an unrooted neighbour-joining phylogenetic tree was constructed using FigTree software V1.4.3, with no presumption of an evolutionary hierarchy. The construction of this tree involved 1000 bootstrap replicates to enhance its robustness.

Evaluation of the variations present within and between the pigeon pea genotypes was investigated using the AMOVA method using GenAlEx V6.503 software.

### 4.14. Principal Coordinate Analysis

Principal coordinate analysis (PCoA) was performed using GenAlex V6.503 software [120] to visualize the relationships among the pigeon pea genotypes.

### 4.15. Statistical Analysis

R Studio was utilized to conduct correlation analysis and the generated graphs. The data used for this analysis encompassed physiological readings collected over three days, consolidated into distinct data sets. For instance, control readings for three days (D1, D2, and D3) were merged into one “control” data set. Similarly, data for 25% FC and 50% FC were grouped into separate data sets. The regression analyses in this report are structured by genotype and ordered as control, 25% FC, and 50% FC, allowing for systematic data comparison across treatments.

For the collected physiological and biochemical parameters, the data underwent analysis of variance (ANOVA) using the F test at a significance level of 5%. Post hoc comparisons of means were conducted using Scott–Knott’s test (*p* ≤ 0.05) with the Sisvar software Version 1.0. The clustering analyses were designed using Microsoft Excel software 365, and the radar plot graphs were plotted through Sigma Plot 11.0 software (SPSS Inc., San Jose, CA, USA). Additionally, principal coordinate analysis (PCoA) was executed on the data sets using GenAlex V6.503 software [120].

## 5. Conclusions

The identification of new genetic resources that are tolerant to drought-stressed conditions is crucial. In addition, attention has to be given to identifying suitable physiological and biochemical markers that can be employed to distinguish the tolerant and susceptible genotypes. This study identified LM and KAT as the most drought-resistant genotypes, characterized by the lowest decrease in % dry weight, a high ability to maintain water content, a higher concentration of osmo-protectants with higher antioxidant enzymes, and a lower ROS accumulation, which contributed to the preservation of soluble sugars under water stress conditions. These genotypes were characterized by superior tolerance to water stress conditions, and emerged as promising candidates for the development of drought-tolerant pigeon pea genotypes. The robust performance of genotypes under adverse conditions positions them as potential parents in breeding programs that could contribute to enhanced drought resilience in the pigeon pea. High levels of genetic diversity among the eight pigeon pea genotypes were shown by SCoT markers. Additionally, the SCoT markers successfully identified molecular markers between the different genotypes and labelled each genotype with distinct bands. The high genetic diversity is crucial for the adaptability and resilience of pigeon pea genotypes in the face of changing environmental conditions. Future studies on gene expression profiling of drought-tolerant pigeon pea genotypes need to be performed to further explore he genetic traits of the selected drought-tolerant genotypes.

## Figures and Tables

**Table 4:** Polymorphism parameters in pigeon pea genotypes identified by SCoT markers.

No. of Primers	Primer Code	No. of Amplified Bands	Polymorphic Bands	% Polymorphic Bands	Unique Bands	Monomorphic Bands	He	PIC	Rp	EMR	MI	D
1	SCoT 2	5	5	100	0	0	0.47	0.36	0.75	0.63	0.04	0.64
2	SCoT 3	44	38	86.4	6	0	0.41	0.33	11	5.5	0.02	0.92
3	SCoT 4	52	43	82.7	1	8	0.50	0.37	7.5	6.5	0.03	0.79
4	SCoT 6	41	40	85.1	1	0	0.50	0.37	8.25	5.13	0.03	0.79
5	SCoT 7	48	47	97.9	1	0	0.50	0.38	8	6	0.03	0.75
6	SCoT 8	26	21	80.8	5	0	0.42	0.33	5.5	3.25	0.02	0.92
7	SCoT 10	24	0	0	0	24	0	0	0	3	0	0
8	SCoT 11	24	23	95.8	1	0	0.5	0.37	3.25	3.13	0.03	0.73
9	SCoT 14	54	29	53.7	1	24	0.50	0.37	6	6.75	0.04	0.69
10	SCoT 15	79	52	65.8	3	24	0.50	0.37	9.25	9.88	0.03	0.70
11	SCoT 16	35	18	51.4	1	16	0.47	0.36	4.75	5.63	0.04	0.61
12	SCoT 20	8	4	50	4	0	0.32	0.27	2	1	0.01	0.96
13	SCoT 21	50	36	72	6	8	0.5	0.37	4.5	6.25	0.03	0.77
14	SCoT 23	96	19	19.8	5	72	0.42	0.33	2.5	12	0.04	0.50
15	SCoT 24	46	45	97.8	1	0	0.49	0.37	5.5	5.75	0.04	0.67
16	SCoT 29	4	4	0	0	0	0.5	0.38	1	0.5	0.03	0.79
17	SCoT 30	52	42	80.8	2	8	0.5	0.37	5.5	6.25	0.03	0.73
18	SCoT 32	36	26	72.2	2	8	0.5	0.37	5	4.5	0.03	0.8
19	SCoT 34	80	5	6.25	3	72	0.35	0.29	1.5	10	0.03	0.41
20	SCoT 35	44	44	100	0	0	0.43	0.34	4	5.5	0.04	0.53
Mean		41.2	27.05	64.92	2.15	14.4	0.44	0.34	4.79	5.36	0.023	0.69

Number of amplified bands (NAB), monomorphic (M), polymorphic (P), percentage of polymorphic (%P), effective multiplex ratio (EMR), marker index (MI), and discriminating power (D).

**Table 5:** Pairwise genetic similarity matrix of eight pigeon pea accessions as revealed by SCoT primers based on Jaccard’s coefficient.

	P1	P2	P3	SM	MM	LM	KAT	P9
P1	1	0.480	0.479	0.429	0.455	0.485	0.430	0.336
P2		1	0.544	0.534	0.537	0.555	0.399	0.349
P3			1	0.595	0.560	0.586	0.506	0.433
SM				1	0.676	0.621	0.449	0.470
MM					1	0.656	0.435	0.436
LM						1	0.535	0.396
KAT							1	0.485
P9								1

**Table 6:** Analysis of molecular variance utilizing SCoT markers in the eight pigeon pea genotypes.

Source	df	SS	MS	Estimated Variance	Percentage	*p* Value
Among population	2	101.375	50.688	7.652	20%	<0.001
Within population	5	153.000	30.600	30.600	80%	<0.001
Total	7	254.375		38.252	100%	

**Table 7:** Sequence, %GC content, amplicons size range, and annealing temperatures of SCoT primers used in this study.

Primer Name	Sequence	%GC Content	Amplicons Size Range (bp)	Annealing Temperature (°C)
SCoT-2	5′-CAACAATGGCTACCACCC-3′	55.6	750	50
SCoT-3	5′-CAACAATGGCTACCACCG-3′	55.6	300–2200	49
ScoT-4	5′-CAACAATGGCTACCACCT-3′	50	250–2000	49
SCoT-6	5′-CAACAATGGCTACCACGC-3′	50	500–3000	48
SCoT-7	5′-CAACAATGGCTACCACGG-3′	55.6	400–2000	48
SCoT-8	5′-CAACAATGGCTACCACGT-3′	50	400–3000	48
SCoT-10	5′-CAACAATGGCTACCAGCC-3′	55.6	700–1250	52
SCoT -11	5′-AAGCAATGGCTACCACCA-3′	50	300–1000	51
SCoT-14	5′-ACGACATGGCGACCACGC-3′	66.7	250–1500	56
SCoT-15	5′-ACGACATGGCGACCGCGA-3′	61.1	200–2500	52
SCoT-16	5′-ACCATGGCTACCACCGAC-3′	61.1	300–1400	50
SCoT-20	5′-ACCATGGCTACCACCGCG-3′	66.7	600–1300	54
SCoT-21	5′-ACGACATGGCGACCCACA-3′	61.1	150–2500	53.5
SCoT-23	5′-CACCATGGCTACCACCAG-3′	61.1	250–1750	51
SCoT-24	5′-CACCATGGCTACCACCAT-3′	55.6	250–1500	52
SCoT-29	5′-CCATGGCTACCACCGGCC-3′	72.2	500	50
SCoT-30	5′-CCATGGCTACCACCGGCG-3′	72.2	500–2500	54
SCoT-32	5′-CCATGGCTACCACCGCAC-3′	66.7	300–2000	52
SCoT-34	5′-ACCATGGCTACCACCGCA-3′	61.1	250–1500	51
SCoT-35	5′-CATGGCTACCACCGGCCC-3′	72.2	150–800	51

## Data Availability

Data are contained within the article.

## Supplementary Materials

The following supporting information can be downloaded at: https://www.mdpi.com/article/10.3390/plants13223228/s1, Figure S1: Electrophoresis gel image of PCR amplicons using SCoT markers for pigeon pea genotypes; Table S1: Physiological and biochemical assays relative to changes due to drought treatments in pigeon pea cultivars. Table S2: Mean-square values of analysis of variance (ANOVA) physiological traits for eight pigeon pea genotypes; Table S3: Genetic characteristics of eight pigeon pea genotypes as revealed by SCoT markers using PowerMarker v3.25.

## References

[1] Fatokimi E.O., Tanimonure V.A. (2021). Analysis of the current situation and future outlooks for pigeon pea (*Cajanus cajan*) production in Oyo State, Nigeria: A Markov Chain model approach. J. Agric. Food Res..

[2] Mergeai G., Kimani P., Mwang’ombe A., Olubayo F., Smith C., Audi P., Le Roi A. (2001). Survey of pigeon pea production systems, utilisation and marketing in semi-arid lands of Kenya. Biotechnol. Agron. Soci. Environ..

[3] Saxena K.B. (2008). Genetic improvement of pigeon pea—A review. Trop. Plant Biol..

[4] Saxena K.B., Ravishankar K., Vijaya Kumar R., Sreejith K.P., Srivastava R.K. (2010). Vegetable Pigeon Pea—A High Protein Food for All Ages.

[5] Mula M.G., Saxena K.B. (2010). Lifting the Level of Awareness on Pigeon Pea—A Global Perspective.

[6] Saxena K.B., Vijaya K.R., Sultana R. (2010). Quality nutrition through pigeon pea—A review. Health.

[7] Pal D., Mishra P., Sachan N., Ghosh A.K. (2011). Biological activities and medicinal properties of *Cajanus cajan* (L.) Millsp. J. Adv. Pharm. Technol. Res..

[8] Høgh-Jensen H. (2011). To meet future food demands we need to change from annual grain legumes to multipurpose semi-perennial legumes. Food Production-Approaches, Challenges and Tasks.

[9] Lose S.J., Hilger T.H., Leihner D.E., Kroschel J. (2003). Cassava, maize and tree root development as affected by various agroforestry and cropping systems in Bénin, West Africa. Agric. Ecosyst. Environ..

[10] Odeny D.A. (2007). The potential of pigeon pea (*Cajanus cajan* (L.) Millsp.) in Africa. Nat. Resour. Forum.

[11] Mutegi J., Zingore S. (2014). Closing crop yield gaps in sub-Saharan Africa through integrated soil fertility management. ISFM Pol. High..

[12] Choudhary A.K., Sultana R., Vales M.I., Saxena K.B., Kumar R.R., Ratnakumar P. (2018). Integrated physiological and molecular approaches to improvement of abiotic stress tolerance in two pulse crops of the semi-arid tropics. Crop J..

[13] Saxena K.B., Choudhary A.K., Saxena R.K., Varshney R.K. (2018). Breeding pigeon pea cultivars for intercropping: Synthesis and strategies. Breed. Sci..

[14] Gill S.S., Tuteja N. (2010). Reactive oxygen species and antioxidant machinery in abiotic stress tolerance in crop plants. Plant Physiol. Biochem..

[15] Laxa M., Liebthal M., Telman W., Chibani K., Dietz K.J. (2019). The role of the plant antioxidant system in drought tolerance. Antioxidants.

[16] Sharma A., Kumar V., Shahzad B., Ramakrishnan M., Singh Sidhu G.P., Bali A.S., Zheng B. (2020). Photosynthetic response of plants under different abiotic stresses: A review. J. Plant Growth Regul..

[17] Anjum N.A., Sofo A., Scopa A., Roychoudhury A., Gill S.S., Iqbal M., Lukatkin A.S., Pereira E., Duarte A.C., Ahmad I. (2015). Lipids and proteins—Major targets of oxidative modifications in abiotic stressed plants. Environ. Sci. Pollut. Res. Int..

[18] Mittler R. (2002). Oxidative stress, antioxidants and stress tolerance. Trends Plant Sci..

[19] Hasanuzzaman M., Nahar K., Gill S.S., Fujita M., Tuteja N., Gill S.S. (2013). Drought stress responses in plants, oxidative stress, and antioxidant defence. Climate Change and Plant Abiotic Stress Tolerance.

[20] Choudhury F.K., Rivero R.M., Blumwald E., Mittler R. (2017). Reactive oxygen species, abiotic stress and stress combination. Plant J..

[21] Lesk C., Rowhani P., Ramankutty N. (2016). Influence of extreme weather disasters on global crop production. Nature.

[22] Kaur H., Kohli S.K., Khanna K., Bhardwaj R. (2021). Scrutinising the impact of water deficit in plants: Transcriptional regulation, signalling, photosynthetic efficacy, and management. Physiol. Plant.

[23] Lipiec J., Doussan C., Nosalewicz A., Kondracka K. (2013). Effect of drought and heat stresses on plant growth and yield: A review. Int. Agrophys..

[24] Gupta A., Rico-Medina A., Caño-Delgado A.I. (2020). The physiology of plant responses to drought. Science.

[25] Farooq M., Wahid A., Kobayashi N.S.M.A., Fujita D.B.S.M.A., Basra S.M.A. (2009). Plant drought stress: Effects, mechanisms and management. Sustainable Agriculture.

[26] Govindaraj M., Rai K.N., Kanatti A., Upadhyaya H.D., Shivade H., Rao A.S. (2020). Exploring the genetic variability and diversity of pearl millet core collection germplasm for grain nutritional traits improvement. Sci. Rep..

[27] Kinhoégbè G., Djèdatin G., Saxena R.K., Chitikineni A., Bajaj P., Molla J., Varshney R.K. (2022). Genetic diversity and population structure of pigeon pea (*Cajanus cajan* [L.] Millspaugh) landraces grown in Benin revealed by Genotyping-By-Sequencing. PLoS ONE.

[28] Bhandari H.R., Bhanu A.N., Srivastava K., Singh M.N., Shreya H.A. (2017). Assessment of genetic diversity in crop plants-an overview. Adv. Plants Agric. Res..

[29] Perez-de-Castro A.M., Vilanova S., Cañizares J., Pascual L., Blanca M.J., Diez M.J., Picó B. (2012). Application of genomic tools in plant breeding. Curr. Genom..

[30] Nandini B., Venkatesh, Reddy U.G., Mallikarjuna B.P., Manu B., Vaijayanthi P.V., Kumar C.J., Manjunath L., Chakraborti D. (2022). Genomic Design for Abiotic Stress Resistance in Pigeon pea. Genomic Designing for Abiotic Stress Resistant Pulse Crops.

[31] Satheesh Naik S.J., Bohra A., Singh I.P., Tiwari A. (2022). Pigeon pea Breeding. Fundamentals of Field Crop Breeding.

[32] Pazhamala L., Saxena R.K., Singh V.K., Sameerkumar C.V., Kumar V., Sinha P., Patel K., Obala J., Kaoneka S.R., Tongoona P. (2015). Genomics-assisted breeding for boosting crop improvement in pigeon pea (*Cajanus cajan*). Front. Plant Sci..

[33] Varshney R.K., Penmetsa R.V., Dutta S., Kulwal P.L., Saxena R.K., Datta S., Cook D.R. (2010). Pigeon pea genomics initiative (PGI): An international effort to improve crop productivity of pigeon pea (*Cajanus cajan* L.). Mol. Breed..

[34] Rathinam M., Mishra P., Vasudevan M., Budhwar R., Mahato A., Prabha A.L., Sreevathsa R. (2019). Comparative transcriptome analysis of pigeon pea, *Cajanus cajan* (L.) and one of its wild relatives *Cajanus platycarpus* (Benth.) Maesen. PLoS ONE.

[35] Upadhyaya H.D., Dwivedi S.L., Ambrose M., Ellis N., Berger J., Smýkal P., Gowda C.L.L. (2011). Legume genetic resources: Management, diversity assessment, and utilisation in crop improvement. Euphytica.

[36] Pandey M.K., Roorkiwal M., Singh V.K., Ramalingam A., Kudapa H., Thudi M., Varshney R.K. (2016). Emerging genomic tools for legume breeding: Current status and future prospects. Front Plant Sci..

[37] Varshney R.K., Roorkiwal M., Nguyen T. (2013). Legume genomics: From genomic resources to molecular breeding. Plant Genome.

[38] Rai M.K. (2023). Start codon targeted (SCoT) polymorphism marker in plant genome analysis: Current status and prospects. Planta.

[39] Collard B.C., Mackill D.J. (2009). Start Codon Targeted (SCoT) Polymorphism: A Simple, Novel DNA Marker Technique for Generating Gene-Targeted Markers in Plants. Plant Mol. Biol. Rep..

[40] Mahjbi A., Baraket G., Oueslati A., Salhi-Hannachi A. (2015). Start Codon Targeted (SCoT) markers provide new insights into the genetic diversity analysis and characterisation of Tunisian Citrus species. Biochem. Syst. Ecol..

[41] Pakseresht F., Talebi R., Karami E. (2013). Comparative assessment of ISSR, DAMD and SCoT markers for evaluation of genetic diversity and conservation of landrace chickpea (*Cicer arietinum* L.) genotypes collected from northwest of Iran. Physiol. Mol. Biol. Plants.

[42] Gowayed S.M., Abd El-Moneim D. (2021). Detection of genetic divergence among some wheat (*Triticum aestivum* L.) genotypes using molecular and biochemical indicators under salinity stress. PLoS ONE.

[43] Guo D.L., Zhang J.Y., Liu C.H. (2012). Genetic diversity in some grape varieties revealed by SCoT analyses. Mol. Biol. Rep..

[44] Akello M., Nyaboga E.N., Badji A., Rubahaiyo P. (2023). Deciphering the morph-physiological and biochemical responses in *Lablab purpureus* (L.) sweet sedlings to water stress. S. Afr. J. Bot..

[45] Henriques F.S. (2009). Leaf chlorophyll fluorescence: Background and fundamentals for plant biologists. Bot. Rev..

[46] Magdaong N.C.M., Blankenship R.E. (2018). Photoprotective, excited-state quenching mechanisms in diverse photosynthetic organisms. J. Biol. Chem..

[47] Tsai H.J., Shao K.H., Chan M.T., Cheng C.P., Yeh K.W., Oelmüller R., Wang S.J. (2020). Piriformospora indica symbiosis improves water stress tolerance of rice through regulating stomata behaviour and ROS scavenging systems. Plant Signal. Behav..

[48] Ruban A.V. (2016). Nonphotochemical chlorophyll fluorescence quenching: Mechanism and effectiveness in protecting plants from photodamage. Plant Physiol..

[49] Chauhan J., Singh P., Choyal P., Mishra U.N., Saha D., Kumar R., Singhal R.K. (2023). Plant photosynthesis under abiotic stresses: Damages, adaptive, and signalling mechanisms. Plant Stress.

[50] Kramer D.M., Johnson G., Kiirats O., Edwards G.E. (2004). New fluorescence parameters for the determination of QA redox state and excitation energy fluxes. Photosynth. Res..

[51] Hussain M.I., Reigosa M.J. (2011). A chlorophyll fluorescence analysis of photosynthetic efficiency, quantum yield and photon energy dissipation in PSII antennae of *Lactuca sativa* L. leaves exposed to cinnamic acid. Plant Physiol. Biochem..

[52] Ochola C.A., Ngugi M.P., Nyaboga E.N., Njarui D.M.G. (2024). Morpho-physiological and yield traits for selection of drought tolerant Urochloa grass ecotypes. AoB PLANTS.

[53] Colom M.R., Vazzana C. (2003). Photosynthesis and PSII functionality of drought-resistant and drought-sensitive weeping lovegrass plants. Environ. Exp. Bot..

[54] Santos A.S., Amorim E.P., Ferreira C.F., Pirovani C.P. (2018). Water stress in *Musa* spp.: A systematic review. PLoS ONE.

[55] Arjenaki F.G., Jabbari R., Morshedi A. (2012). Evaluation of drought stress on relative water content, chlorophyll content and mineral elements of wheat (*Triticum aestivum* L.) varieties. Int. J. Agric. Crop Sci..

[56] Gutierrez J.C., Lopez M., Leidi E.O. (1998). Drought Susceptibility Index as an indicator of genotypic drought tolerance in upland cotton. Proc. World Cotton Res. Conf. II.

[57] Barik S.R., Pandit E., Pradhan S.K., Singh S., Swain P., Mohapatra T. (2018). QTL mapping for relative water content trait at reproductive stage drought stress in rice. Indian J. Genet. Plant Breed.

[58] Sapes G., Sala A. (2021). Relative water content consistently predicts drought mortality risk in seedling populations with different morphology, physiology and times to death. Plant Cell Environ..

[59] Hasheminasab H., Farshadfar E., Yaghotipoor A. (2013). Investigation of water retention capacity (WRC) as a new physiological indicator related to plant water status for screening drought tolerant genotypes in wheat. J. Biodivers Environ. Sci..

[60] Bayoumi T.Y., Eid M.H., Metwali E.M. (2008). Application of physiological and biochemical indices as a screening technique for drought tolerance in wheat genotypes. Afr. J. Biotechnol..

[61] Jia X., Zhang Z., Liu Y., Liu B. (2016). Effects of drought stress on the photosynthetic and physiological characteristics of maize seedlings. Agric Sci. Technol..

[62] Niu L., Yuan H., Sun X., Zhu M., Dong M. (2019). Effects of drought stress on plant growth and osmotic adjustment in leaves of apple seedlings. J. Fruit Sci..

[63] Suzuki N., Rivero R.M., Shulaev V., Blumwald E., Mittler R. (2012). Abiotic and biotic stress combinations. New Phytol..

[64] Das K., Roychoudhury A. (2014). Reactive oxygen species (ROS) and response of antioxidants as ROS-scavengers during environmental stress in plants. Front. Environ. Sci..

[65] Hossain M.A., Bhattacharjee S., Armin S.M., Qian P., Xin W., Li H.Y., Tran L.S.P. (2015). Hydrogen peroxide priming modulates abiotic oxidative stress tolerance: Insights from ROS detoxification and scavenging. Front. Plant Sci..

[66] Liu F., Xu W., Wei W., Zhou Q., Yang L., Chen T., Chen W. (2019). Peroxidase activity of roots is involved in the drought tolerance of peanut. Crop J..

[67] Chugh V., Kaur N., Grewal M.S., Gupta A.K. (2013). Differential antioxidative response of tolerant and sensitive maize (*Zea mays* L.) genotypes to drought stress at reproductive stage. Indian J. Biochem. Biophys..

[68] Chaves M.M., Pereira J.S., Maroco J., Rodrigues M.L., Ricardo C.P., Osorio M.L., Carvalho I., Faria T., Pinheiro C. (2002). How plants cope with water stress in the field? Photosynthesis and growth. Ann. Bot..

[69] Rajput V.D., Harish S.R.K., Verma K.K., Sharma L., Quiroz-Figueroa F.R., Meena M., Gour V.S., Minkina T., Sushkova S., Mandzhieva S. (2021). Recent developments in enzymatic antioxidant defence mechanism in plants with special reference to abiotic stress. Biology.

[70] Mittler R., Zilinskas B.A. (1994). Regulation of pea cytosolic ascorbate peroxidase and other antioxidant enzymes during the progression of drought stress and following recovery from drought. Plant J..

[71] Esfandiari E., Shakiba M.R., Mahboob S.A., Alyari H., Toorchi M. (2007). Water stress, antioxidant enzyme activity and lipid peroxidation in wheat seedling. J. Food Agric. Environ..

[72] Murshed R., Lopez-Lauri F., Sallanon H. (2013). Effect of water stress on antioxidant systems and oxidative parameters in fruits of tomato (*Solanum lycopersicon* L., cv. Micro-tom). Physiol. Mol. Biol. Plants.

[73] Plazas M., Nguyen H.T., González-Orenga S., Fita A., Vicente O., Prohens J., Boscaiu M. (2019). Comparative analysis of the responses to water stress in eggplant (*Solanum melongena*) cultivars. Plant Physiol. Biochem..

[74] Rahimi M., Kordrostami M., Mortezavi M. (2019). Evaluation of tea (*Camellia sinensis* L.) biochemical traits in normal and drought stress conditions to identify drought tolerant clones. Physiol. Mol. Biol. Plants..

[75] Dixon R.A., Paiva N.L. (1995). Stress-induced phenylpropanoid metabolism. Plant Cell.

[76] Yadav V., Wang Z., Wei C., Amo A., Ahmed B., Yang X., Zhang X. (2020). Phenylpropanoid pathway engineering: An emerging approach towards plant defence. Pathogens.

[77] Vogt T. (2010). Phenylpropanoid biosynthesis. Mol. Plant.

[78] Chaves M.M., Flexas J., Pinheiro C. (2003). Photosynthesis under drought and salt stress: Regulation mechanisms from whole plant to cell. Ann. Bot..

[79] Pandey K., Kumar R.S., Prasad P., Pande V., Trivedi P.K., Shirke P.A. (2022). Coordinated regulation of photosynthesis and sugar metabolism in guar increases tolerance to drought. Environ. Exp. Bot..

[80] Nunes-Nesi A., Fernie A.R., Stitt M. (2010). Metabolic and signalling aspects underpinning the regulation of plant carbon nitrogen interactions. Mol. Plant.

[81] Sperdouli I., Moustakas M. (2012). Interaction of proline, sugars, and anthocyanins during photosynthetic acclimation of Arabidopsis thaliana to drought stress. J. Plant Physiol..

[82] Pinheiro C., Chaves M.M. (2011). Photosynthesis and drought: Can we make metabolic connections from available data?. J. Exp. Bot..

[83] Mansour M.M.F. (2000). Nitrogen containing compounds and adaptation of plants to salinity stress. Biol. Plant..

[84] Parvaiz A., Satyawati S. (2008). Salt stress and phyto-biochemical responses of plants—A review. Plant Soil Environ..

[85] Sharma P., Dubey R.S., Saed-Moucheshi A. (2019). Protein synthesis by plants under stressful conditions. Handbook of Plant and Crop Stress.

[86] Xin L., Zheng H., Yang Z., Guo J., Liu T., Sun L., Guo L. (2018). Physiological and proteomic analysis of maize seedling response to water deficiency stress. J. Plant Physiol..

[87] Heinemann B., Künzler P., Eubel H., Braun H.P., Hildebrandt T.M. (2021). Estimating the number of protein molecules in a plant cell: Protein and amino acid homeostasis during drought. Plant Physiol..

[88] Khan N., Ali S., Zandi P., Mehmood A., Ullah S., Ikram M., Babar M.A. (2020). Role of sugars, amino acids and organic acids in improving plant abiotic stress tolerance. Pak. J. Bot..

[89] Khalilzadeh R., Saeidi G., Tavakoli M. (2019). Evaluation of drought tolerance in chickpea genotypes based on protein, proline, carbohydrate and yield. Plant Prod. Technol..

[90] Chen T.H., Murata N. (2002). Enhancement of tolerance of abiotic stress by metabolic engineering of betaines and other compatible solutes. Curr. Opin. Plant Biol..

[91] Vendruscolo E.C.G., Schuster I., Pileggi M., Scapim C.A., Molinari H.B.C., Marur C.J., Vieira L.G.E. (2007). Stress-induced synthesis of proline confers tolerance to water deficit in transgenic wheat. J. Plant Physiol..

[92] Zhu J.K. (2002). Salt and drought stress signal transduction in plants. Annu. Rev. Plant Biol..

[93] de Freitas P.A.F., de Carvalho H.H., Costa J.H., Miranda R.D.S., Saraiva K.D.D.C., de Oliveira F.D.B., Gomes-Filho E. (2019). Salt acclimation in sorghum plants by exogenous proline: Physiological and biochemical changes and regulation of proline metabolism. Plant Cell Rep..

[94] Chutipaijit S., Cha-Um S., Sompornpailin K. (2009). Differential accumulations of proline and flavonoids in indica rice varieties against salinity. Pak. J. Bot..

[95] Ahmed M., Qadir G., Shaheen F.A., Aslam M.A. (2017). Response of proline accumulation in bread wheat (*Triticum aestivum* L.) under rainfed conditions. J. Agric. Meteorol..

[96] Sun C., Li X., Hu Y., Zhao P., Xu T., Sun J., Gao X. (2015). Proline, sugars, and antioxidant enzymes respond to drought stress in the leaves of strawberry plants. Hortic. Sci. Technol..

[97] Alexieva V., Sergiev I., Mapelli S., Karanov E. (2001). The effect of drought and ultraviolet radiation on growth and stress markers in pea and wheat. Plant Cell Environ..

[98] Yamada M., Morishita H., Urano K., Shiozaki N., Yamaguchi-Shinozaki K., Shinozaki K., Yoshiba Y. (2005). Effects of free proline accumulation in petunias under drought stress. J. Exp. Bot..

[99] Eltaher S., Sallam A., Belamkar V., Emara H.A., Nower A.A., Salem K.F., Baenziger P.S. (2018). Genetic diversity and population structure of F3:6 Nebraska winter wheat genotypes using genotyping-by-sequencing. Front Genet..

[100] Nkhata W., Shimelis H., Melis R., Chirwa R., Mzengeza T., Mathew I., Shayanowako A. (2020). Population structure and genetic diversity analyses of common bean germplasm collections of East and Southern Africa using morphological traits and high-density SNP markers. PLoS ONE.

[101] Kimaro D., Melis R., Sibiya J., Shimelis H., Shayanowako A. (2020). Analysis of genetic diversity and population structure of pigeon pea [*Cajanus cajan* (L.) Millsp] accessions using SSR markers. Plants.

[102] Roldan-Ruiz I., Dendauw J., Van Bockstaele E., Depicker A., De Loose M. (2000). AFLP markers reveal high polymorphic rates in ryegrasses (*Lolium* spp.). Mol. Breed..

[103] Chen W., Hou L., Zhang Z., Pang X., Li Y. (2017). Genetic diversity, population structure, and linkage disequilibrium of a core collection of *Ziziphus jujuba* assessed with genome-wide SNPs developed by genotyping-by-sequencing and SSR markers. Front Plant Sci..

[104] Kassa M.T., Penmetsa R.V., Carrasquilla-Garcia N., Sarma B.K., Datta S., Upadhyaya H.D., Varshney R.K., von Wettberg E.J., Cook D.R. (2012). Genetic patterns of domestication in pigeon pea (*Cajanus cajan* (L.) Millsp.) and wild Cajanus relatives. PLoS ONE.

[105] Vincent H., Wiersema J., Kell S., Fielder H., Dobbie S., Castañeda-Álvarez N.P., Maxted N. (2013). A prioritised crop wild relative inventory to help underpin global food security. Biol. Conserv..

[106] Yang S., Pang W., Ash G., Harper J., Carling J., Wenzl P., Kilian A. (2006). Low level of genetic diversity in cultivated pigeon pea compared to its wild relatives is revealed by diversity arrays technology. Theor. Appl. Genet..

[107] Yadav P., Saxena K.B., Hingane A., Kumar C.V., Kandalkar V.S., Varshney R.K., Saxena R.K. (2019). An “Axiom Cajanus SNP Array” based high density genetic map and QTL mapping for high-selfing flower and seed quality traits in pigeon pea. BMC Genom..

[108] Saxena K.B., Singh L., Gupta M.D. (1990). Variation for natural out-crossing in pigeon pea. Euphytica.

[109] Sibomana I.C., Aguyoh J.N., Opiyo A.M. (2013). Water stress affects growth and yield of container grown tomato (*Lycopersicon esculentum* Mill) plants. Gjbb.

[110] Mullan D., Pietragalla J. (2012). Leaf relative water content. Physiological Breeding II: A Field Guide to Wheat Phenotyping.

[111] Shin L.J., Lo J.C., Yeh K.C. (2012). Copper chaperone antioxidant protein1 is essential for copper homeostasis. Plant Physiol..

[112] Velikova V., Yordanov I., Edreva A.J. (2000). Oxidative stress and some antioxidant systems in acid rain-treated bean plants: Protective role of exogenous polyamines. Plant Sci..

[113] Nakano Y., Asada K. (1981). Hydrogen peroxide is scavenged by ascorbate-specific peroxidase in spinach chloroplasts. Plant Cell Physiol..

[114] Cakmak I., Strbac D., Marschner H. (1993). Activities of hydrogen peroxide-scavenging enzymes in germinating wheat seeds. J. Exp. Bot..

[115] Chen W., Gao L., Song L., Sommerfeld M., Hu Q. (2023). An improved phenol-sulfuric acid method for the quantitative measurement of total carbohydrates in algal biomass. Algal Res..

[116] Ainsworth E.A., Gillespie K.M. (2007). Estimation of total phenolic content and other oxidation substrates in plant tissues using Folin–Ciocalteu reagent. Nat. Protoc..

[117] Bradford M.M. (1976). A rapid and sensitive method for the quantitation of microgram quantities of protein utilising the principle of protein-dye binding. Anal. Biochem..

[118] Huang S., Wu Y. (2010). Quantitative determination of total free-amino acid in *Nervilia fordii* (Hance) Schltr. by ninhydrin colorimetric method. Chin. J. Inf. Tradit. Chin. Med..

[119] Satya P., Karan M., Jana S., Mitra S., Sharma A., Karmakar P.G., Ray D.P. (2015). Start codon targeted (SCoT) polymorphism reveals genetic diversity in wild and domesticated populations of ramie (*Boehmeria nivea* L. Gaudich.), a premium textile fibre producing species. Meta Gene.

[120] Smouse R.P.P., Peakall R. (2012). GenAlEx 6.5: Genetic analysis in Excel. Population genetic software for teaching and research—An update. Bioinformatics.

[121] Pritchard J.K., Stephens M., Donnelly P. (2000). Inference of population structure using multilocus genotype data. Genetics.

