# Supplementary material for: Integrating Morpho-Physiological, Biochemical, and Molecular Genotyping for Selection of Drought-Tolerant Pigeon Pea (Cajanus cajan L.) Genotypes at Seedling Stage
Source: Plants (Basel). 2024 Nov 16;13(22):3228. doi: 10.3390/plants13223228 (PMC11598755; doi:10.3390/plants13223228)
Supplement: Supplementary file 1 [file plants-13-03228-s001.zip › plants-3088951-supplementary.pdf]

Supplementary Material

Integrating Morpho-physiological, Biochemical and Molecular Genotyping for Selection of Drought Tolerant Pigeon Pea (*Cajanus cajan* L.) Genotypes at Seedling Stage

Benjamin O. Ouma[1], Kenneth Mburu[2,*], Geoffrey K. Kirui[3], Edward K. Muge[1], and Evans N. Nyaboga[1]

[1] Department of Biochemistry, University of Nairobi, P.O. Box 30197, Nairobi 00100, Kenya
[2] Department of Life Sciences, South Eastern Kenya University, P.O. Box 170, Kitui 90200, Kenya
[3] Department of Biology, University of Nairobi, P.O. Box 30197, Nairobi 00100, Kenya
* Correspondence: kmburu@seku.ac.ke

Supplementary Tables

Supplementary Table S1. Physiological and biochemical assays relative to changes due to drought treatments in pigeon pea genotypes. FC – 100% field capacity, 50%FC – 50% field capacity, 25%FC – 25% field capacity. Genotypes: P1, P2, P3, SM, MM, LM, KAT and P9.

	Genotypes																							
	P1			P2			P3			SM			MM			LM			KAT			P9		
Treatment	FC	50%FC	25%FC	FC	50%FC	25%FC	FC	50%FC	25%FC	FC	50%FC	25%FC	FC	50%FC	25%FC	FC	50%FC	25%FC	FC	50%FC	25%FC	FC	50%FC	25%FC
Trait																								
Phi2	0.60	0.53	0.39	0.59	0.4	0.34	0.5	0.5	0.22	0.6	0.63	0.35	0.59	0.65	0.44	0.6	0.51	0.46	0.61	0.64	0.44	0.64	0.6	0.42
PhiNPQ	0.18	0.25	0.44	0.24	0.46	0.53	0.33	0.33	0.65	0.22	0.16	0.5	0.24	0.15	0.4	0.22	0.29	0.38	0.21	0.16	0.38	0.17	0.22	0.43
PhiNO	0.21	0.21	0.17	0.17	0.14	0.14	0.17	0.17	0.13	0.18	0.2	0.15	0.17	0.2	0.16	0.18	0.2	0.16	0.18	0.2	0.18	0.18	0.18	0.15
NPQt	0.86	1.35	2.80	1.45	3.79	6.7	2.25	2.36	5.16	1.25	0.81	3.71	1.56	0.77	2.91	1.3	1.51	3.63	1.19	0.8	2.13	1.00	1.36	2.89
SPAD	41.93	32.86	33.75	44.46	30.05	29.82	37.67	30.32	34.26	40.83	36.13	37.11	34.04	34.5	37.6	40.72	40.15	38.65	41.55	36.68	35.76	41.28	36.87	35.08
LEF	40.23	39.90	47.40	31.45	40.82	37.3	49.14	44.53	55.2	37.14	38.05	61.71	39.74	28.88	47.3	23.38	77.1	39.77	22.46	28.09	35.94	23.33	37.58	72.8
FvP/FmP	0.72	0.69	0.58	0.67	0.54	0.5	0.63	0.62	0.46	0.69	0.73	0.54	0.67	0.73	0.6	0.69	0.68	0.6	0.69	0.73	0.62	0.71	0.68	0.56
LT	29.42	29.57	31.70	31.37	31.18	35.01	32.89	32.31	37	33.9	32.65	37.41	33.46	32.06	37.06	30.23	32.2	35.55	30.68	33.02	30.75	31.05	33.16	32.25
RWC	80.60	79.21	68.35	74.6	72.24	65.49	73.02	70.16	62.22	79.56	78.3	72.19	89.6	79.48	73.72	86.96	84.13	70.69	86.96	84.96	78.26	81.12	78.87	74.18
TPC	93.34	24.08	23.06	33.72	20.71	38.86	30.01	23.71	17	25.87	21.63	18.14	24.6	17.27	18.02	27.60	19.72	18.82	25.65	17.65	18.39	19.93	17.31	18.87
MDA	1.01	1.12	5.65	2.86	4.86	12.52	1.94	8.46	10.59	2.10	5.13	10.59	2.18	6.64	8.14	1.76	7.41	9.23	2.85	8.42	11.05	1.07	1.54	7.39
SS	0.10	0.10	0.12	0.25	0.13	0.08	0.11	0.07	0.15	0.14	0.09	0.13	0.13	0.08	0.08	0.09	0.15	0.22	0.18	0.10	0.08	0.23	0.15	0.12
Proline	24.44	31.62	138.52	50.16	47.6	188.49	17.95	61.94	35.07	38.28	52.96	154.51	21.26	26.16	124.93	21.23	22.05	149.11	18.96	39.06	137.13	15.89	36.20	111.48
Protein content	109.61	9.83	9.03	19.21	14.49	16.44	21.27	19.51	9.99	45.46	23.15	6.62	51.62	9.17	18.80	3.11	7.96	13.55	30.13	7.34	67.06	23.06	32.56	31.11
TAC	0.04	0.04	0.16	0.03	0.07	0.08	0.12	0.06	0.04	0.02	0.38	0.64	0.01	0.02	0.18	0.01	0.10	0.17	0.05	0.06	0.11	0.03	0.05	0.07
TFAAs	0.44	0.33	0.60	0.31	0.59	0.67	0.17	0.37	0.81	0.04	0.04	0.64	0.59	0.67	0.70	0.24	0.54	0.17	0.36	0.54	0.55	0.22	0.13	0.43
H_2O_2	2.26	2.81	3.67	3.05	3.22	2.67	2.84	2.85	2.91	2.47	2.73	2.85	2.73	2.81	3.05	2.97	3.21	3.08	2.92	2.94	2.76	2.47	2.85	3.02
POD	49.20	32.85	28.92	41.08	38.53	11.99	26.67	35.55	19.47	22.57	29.95	30.45	19.18	25.89	30.98	27.34	32.16	27.41	28.64	27.34	30.52	35.68	30.22	26.14
CAT	1.10	1.58	0.68	0.33	0.45	0.33	0.68	0.58	0.85	0.35	1.25	0.93	0.15	0.68	0.78	0.13	0.53	0.33	0.45	2.13	0.38	1.68	1.15	0.40
APX	6.37	8.39	8.87	85.12	20.18	5.42	4.17	9.88	5.65	6.01	19.70	8.69	1.79	12.80	8.10	67.08	27.08	10.30	4.29	3.39	14.88	2.98	6.19	13.21

Traits: Phi2 – Quantum yield of photosystem II; PhiNPQ – Quantum yield of non-photochemical quenching; PhiNO – Quantum yield of regulated energy dissipation, NPQt – Non-Photochemical Quenching; SPAD – Relative chlorophyll content; LEF – Lateral Electron Flow; FvP/FmP – Photosystem II efficiency; LT – Leaf Temperature; RWC – Relative Water Content; TPC – Total Phenolic Content; MDA – Malondialdehyde content; SS – Soluble sugars; TAC – Total amino acids; TFAA – Total Free Amino Acids; H_2O_2 – Hydrogen peroxide; POD – Total Peroxidase; CAT – Catalase; APX – Ascorbate Peroxidase. For all cases, green colour specifies an increase and red colour denotes a decrease in the analyzed indices, taking the data of 100% as a reference. Grey represents no change.

Supplementary Table S2: Mean square values of analysis of variance (ANOVA) physiological traits for eight pigeon pea genotypes.

Source of variation	DF	Phi2	PhiNPQ	PhiNO	SPAD	LEF	NPQt	FvP/FmP	LT
Genotypes (G)	7	0.029***	0.043***	0.002***	34.42***	403.8*	7.37***	0.017***	20.29***
Water regimen (W)	2	0.304***	0.400***	0.007***	230.72***	1597.6***	41.37***	0.121 ***	62.29 ***
G X W	14	0.009**	0.012**	0.0004*	27.35**	542.9**	1.542*	0.004**	6.39***
Error	48	0.032	0.038	0.0003	11.08	210.3	1.28	0.01	0.11
Total	71								

***, **, *, significant at P<0.001, P<0.01, P<0.05 respectively; Phi2, PhiNPQ, PhiNO, SPAD, Chlorophyll content; LEF, Linear Electron Flow; NPQt, FvP/FmP, maximum quantum efficiency of photosystem 2; LT, Leaf temperature

Supplementary Table S3: Genetic characteristics of 8 pigeon pea genotypes as revealed by SCoT markers using PowerMarker v3.25

Genotype	Major Allele Frequency	Gene Diversity	PIC
P1	0.578	0.488	0.369
P2	0.510	0.500	0.375
P3	0.626	0.468	0.359
SM	0.597	0.481	0.365
MM	0.510	0.500	0.375
LM	0.544	0.496	0.373
KAT	0.515	0.500	0.375
P9	0.534	0.498	0.374
Mean	0.552	0.491	0.371

P1 – Genotype P1, P2 – Genotype P2, P3 – Genotype P3, SM – Short maturing, MM – Medium maturing, LM – Long maturing, KAT – Katumani, P9 – Genotype P9

Supplementary Figures

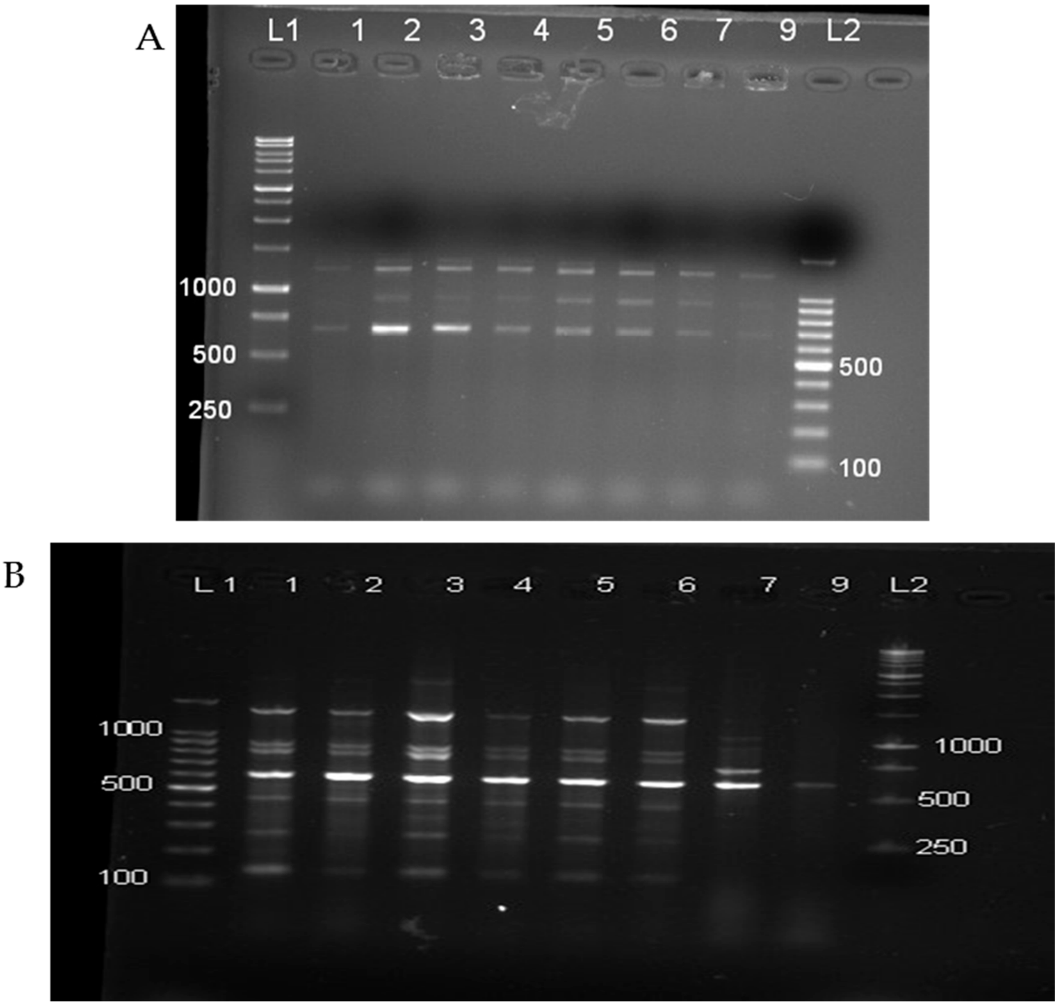

Supplementary Figure S1: Electrophoresis gel image of PCR amplicons using SCoT markers for pigeon pea genotypes. A: SCoT10 and B: SCoT21. L1: GeneRuler 1kb DNA ladder (Promega, Madison -USA), L2: GeneRuler 100 bp DNA ladder (Promega, Madison -USA), and Lanes 1 –7 and 9 are amplified DNA samples of pigeon genotypes (1 - Genotype 1; 2 - Genotype 2; 3 - Genotype 3; 4 - Short maturing (SM) genotype, 5 - Medium maturing (MM) genotype; 6 - Long maturing (LM) genotype, 7 – Katumani (KAT); 9 - Genotype P9.
